# Sex differences in the effects of prematurity and/or low birthweight on neurodevelopmental outcomes: systematic review and meta-analyses

**DOI:** 10.1186/s13293-023-00532-9

**Published:** 2023-07-11

**Authors:** Julian K. Christians, Saboura Ahmadzadeh-Seddeighi, Alishba Bilal, Anastasia Bogdanovic, Rebecca Ho, Estee V. Leung, Megan A. MacGregor, Nolan M. Nadasdy, Gabriella M. Principe

**Affiliations:** 1 Department of Biological Sciences, Simon Fraser University, Burnaby, BC Canada; 2 Centre for Cell Biology, Development and Disease, Simon Fraser University, Burnaby, BC Canada; 3 British Columbia Children’s Hospital Research Institute, Vancouver, BC Canada; 4 Women’s Health Research Institute, BC Women’s Hospital and Health Centre, Vancouver, BC Canada; 5 Department of Molecular Biology and Biochemistry, Simon Fraser University, Burnaby, BC Canada; 6 Department of Pathology and Laboratory Medicine, University of British Columbia, Vancouver, BC Canada; 7 Department of Molecular Oncology, British Columbia Cancer Research Institute, Vancouver, BC Canada; 8 Biomedical Physiology and Kinesiology, Simon Fraser University, Burnaby, BC Canada; 9 Faculty of Health Sciences, Simon Fraser University, Burnaby, BC Canada

**Keywords:** Systematic review, Prematurity, Birthweight, Sex differences, Gender, Cognitive function, Internalizing problems, Externalizing problems, Cognition

## Abstract

**Background:**

Premature birth and/or low birthweight have long-lasting effects on cognition. The purpose of the present systematic review is to examine whether the effects of prematurity and/or low birth weight on neurodevelopmental outcomes differ between males and females.

**Methods:**

Web of Science, Scopus, and Ovid MEDLINE were searched for studies of humans born premature and/or of low birthweight, where neurodevelopmental phenotypes were measured at 1 year of age or older. Studies must have reported outcomes in such a way that it was possible to assess whether effects were greater in one sex than the other. Risk of bias was assessed using both the Newcastle–Ottawa scale and the National Institutes of Health Quality assessment tool for observational cohort and cross-sectional studies.

**Results:**

Seventy-five studies were included for descriptive synthesis, although only 24 presented data in a way that could be extracted for meta-analyses. Meta-analyses found that severe and moderate prematurity/low birthweight impaired cognitive function, and severe prematurity/low birthweight also increased internalizing problem scores. Moderate, but not severe, prematurity/low birthweight significantly increased externalizing problem scores. In no case did effects of prematurity/low birthweight differ between males and females. Heterogeneity among studies was generally high and significant, although age at assessment was not a significant moderator of effect. Descriptive synthesis did not identify an obvious excess or deficiency of male-biased or female-biased effects for any trait category. Individual study quality was generally good, and we found no evidence of publication bias.

**Conclusions:**

We found no evidence that the sexes differ in their susceptibility to the effects of severe or moderate prematurity/low birthweight on cognitive function, internalizing traits or externalizing traits. Result heterogeneity tended to be high, but this reflects that one sex is not consistently more affected than the other. Frequently stated generalizations that one sex is more susceptible to prenatal adversity should be re-evaluated.

**Supplementary Information:**

The online version contains supplementary material available at 10.1186/s13293-023-00532-9.

## Plain Language Summary

Early life environmental conditions and adversities affect health into adulthood. For example, it is well-known that premature birth and low birthweight have long-lasting effects on the development and functioning of the brain, affecting various aspects of academic performance, intelligence, and the risk of behavioural problems including depression, anxiety, aggression, impulsivity, and inattention. However, it is not clear if these effects differ between boys and girls. We searched for studies examining the effects of prematurity and/or of low birthweight on cognitive abilities and behavioural problems in children measured at 1 year of age or older, and identified 75 relevant studies. Combining the results of studies found that prematurity/low birthweight decreased measures of intelligence and increased the incidence of behavioural problems, as expected. However, there was no indication that the effects of prematurity/low birthweight consistently differed between males and females, and there were no specific traits where boys appeared to be more or less susceptible to the effects of prematurity/low birthweight than girls. While sex and gender influence health, and in many cases will influence the effects of early life conditions on health, our study shows that prematurity and low birthweight have similar long-term effects on intelligence and behaviour in boys and girls.

**Supplementary Information:**

The online version contains supplementary material available at 10.1186/s13293-023-00532-9.

## Highlights

- Premature birth and/or low birthweight have long-lasting effects on cognition, but it is not clear if these effects differ between males and females.
- We searched for studies examining the effects of prematurity and/or of low birthweight on neurodevelopmental phenotypes measured at one year of age or older, and identified 75 studies for descriptive synthesis, and 24 for meta-analyses.
- Meta-analyses found that prematurity/low birthweight impaired cognitive function and increased internalizing and externalizing problem scores. However, in no case did effects of prematurity/low birthweight differ between males and females.
- Descriptive synthesis did not identify an obvious excess or deficiency of male-biased or female-biased effects for any type of trait.
- We found no evidence that the sexes differ in their susceptibility to the effects of severe or moderate prematurity/low birthweight on cognitive function, internalizing traits or externalizing traits.

**Supplementary Information:**

The online version contains supplementary material available at 10.1186/s13293-023-00532-9.

## Background

Insults in early life can have far-reaching impacts on health. Numerous systematic reviews have found associations between premature birth and/or low birthweight and cognitive abilities throughout childhood from infancy [1], to preschool age [2–4], to later childhood [5–7] and even into adulthood [8–11]. Even being born late preterm (34–36 weeks [12]) or early term (37–38 weeks [13]) has effects on cognition. Moreover, brain sparing (redistribution of blood flow to the brain) in response to intrauterine growth restriction does not fully protect cognitive abilities [14].

While there is clear and consistent evidence that low birthweight and premature birth have lasting effects on cognition, it is not clear whether males or females may be more susceptible to such effects. Many authors have suggested that males may have greater susceptibility to early life conditions [15–23]. However, with regard to the effects of low birthweight and prematurity, while studies often adjust for effects of sex (i.e., take into account overall differences between males and females), or acknowledge sex as a potentially confounding factor, relatively few assess sex-dependent effects (e.g., whether males are more or less affected by prematurity than females). Moreover, such sex-dependent effects have not been examined in a systematic review, although two studies using meta-regression found that the effect or prematurity or low birthweight was not related to the sex ratio of study participants [24, 25]. Another examined sex-specific effects of nutritional supplements in these populations [26]. A systematic review of the effects of a variety of prenatal stressors on the hypothalamic–pituitary–adrenal axis of the offspring [27] found females more vulnerable. Other work has also suggested that females may be more susceptible to the effect of prenatal adversity on the risk of developing affective problems [28].

The purpose of the present systematic review is to examine whether the effects of prematurity and/or low birth weight on neurodevelopmental outcomes differ between males and females. Because we are interested in exposures that occur prior to birth, we will use the term “sex” for brevity. However, we acknowledge that outcomes are measured after substantial socialization has occurred, and thus will be affected by both sex and gender.

## Methods

We followed the Preferred Reporting Items for Systematic Reviews and Meta-Analyses (PRISMA) guidelines [29]. This study is registered with PROSPERO (CRD42021228814).

### Eligibility criteria, information sources and search strategy

Eligible studies were of humans born premature and/or of low birthweight, where unaffected comparators/controls were also included, i.e., individuals born at term, and/or individuals of normal birth weight. Prematurity was defined as birth prior to 37 weeks of gestation. Low birth weight is defined either in terms of a fixed value (e.g., < 2500 g) or in terms of a percentile (e.g., below the 10th percentile for gestational age). There is substantial heterogeneity with regard to the criteria used to define prematurity and low birthweight, but a meta-analysis has previously supported the use of both gestational age and/or birthweight as inclusion criteria for the study of the effects of prematurity on cognition [30]. The original registration in PROSPERO included exposure to prenatal maternal depression or anxiety or stress, but this was later removed to narrow the scope of the study.

Some studies have suggested increased male vulnerability in long-term behavioral and cognitive outcomes [15, 19, 20, 22, 23], and so we focused on such outcomes measured at 1 year of age or older, including assessments of abilities relating to language (including reading and speech), behaviour, memory, learning, thinking and problem solving. Discrete and continuous outcomes were included. Outcomes that were defined primarily in terms of motor skills, vision, hearing and/or brain morphology (e.g., volumes of brain regions) were excluded.

To be included, studies must have reported outcomes in such a way that it was possible to assess whether the effect of prematurity and/or low birthweight was greater in one sex than the other (e.g., presented separately by sex and/or the statistical interaction between sex and exposure was tested and reported). If differences between the sexes were reported separately for different exposure groups (e.g., differences between males and females were reported separately for preterm and term individuals), but differences between exposures are not reported separately for the sexes, results were not included if it was not possible to assess the latter comparison and extract the relevant data.

Web of Science, Scopus, Ovid MEDLINE, were all searched May 25, 2020, and this search was repeated on May 11, 2022, limiting to publication dates of 2020 or later. The search strategy is provided in Additional file 1.

### Selection process

Three reviewers (AB, JKC, SA) screened non-overlapping sets of titles and abstracts from the first search to exclude studies where the exposure and/or outcome clearly did not meet inclusion criteria (i.e., each title and abstract was screened by one reviewer at this stage). Two reviewers (AB, RH) independently examined the full texts of the remaining studies to assess whether they met eligibility criteria. Disagreements were resolved by a third reviewer (JKC). One reviewer (GMP) screened titles and abstracts from the updated search, and two reviewers (GMP, JKC) independently examined full texts of the remaining studies.

### Data collection and data items

Data collected included type and severity of exposure and comparator group (e.g., < 33 weeks vs term; < 1500 g vs controls), sample size per group, outcome studied, age at which the outcome was studied, the effect of exposure on outcome in males (e.g., means, odds ratios, including standard deviations and/or confidence intervals, as available), the effect of exposure on outcome in females, and the approach to test for sex dependent effects (e.g., presented separately by sex or the statistical interaction between sex and exposure was tested). Where multiple scores were summarized (e.g., multiple measures of cognitive function combined into IQ, or multiple problem scores combined into internalizing and externalizing problems), we extracted only the summary score. Where both continuous scores and proportions above/below a cut off were reported, we extracted only continuous scores. Two reviewers (MAM, NMN) independently extracted data, and disagreements were resolved by a third reviewer (JKC). Where data were provided in figures, they were extracted them WebPlotDigitizer [31].

### Study risk of bias assessment

Studies were assessed for quality and risk of bias by AB and EVL using two scoring systems [32, 33]; criteria are listed in Additional file 2: Table S1. These assessments were only used to assess the quality of the studies, but were not included in meta-analyses.

### Effect measures and synthesis methods

We collected means and standard deviations for continuous outcomes, and odds ratios, relative risks or hazard ratios for discrete outcomes. Upon extraction of data, we found that there was no type of neurodevelopmental outcome for which 5 or more studies reported results for discrete outcomes, and so only studies presenting means for continuous outcomes were included in meta-analyses. Similarly, less than 5 studies reported outcomes for a given type of neurodevelopmental outcome assessed at an age of less than 5 years in such a way that data could be extracted for meta-analyses, and so such studies were excluded from meta-analyses.

Meta-analyses were performed separately on cognitive, internalizing and externalizing traits (see Table 1 for examples of each type of trait). For all traits, values were first scaled, so that the average value for the 4 groups in a given study (exposed males, control males, exposed females, control females) was 100; means and standard deviations were scaled by multiplying all values by a fixed value for a given study. Where a given study measured a trait at multiple ages, or measured multiple traits in the same category (cognitive, internalizing, or externalizing), values were averaged across ages/traits, such that each study contributed only one set of 4 values to the meta-analysis for each category of outcome.

**Table 1:** Categories used to group traits

Category	Examples of traits
Cognitive	Spatial skills, math, problem solving, reasoning, general IQ, full scale IQ, memory, academic competence
Internalizing	Depression, anxiety, emotional problems, neuroticism, OCD symptoms, emotional and peer subscales of Strength and Difficulties Questionnaire [34], social phobia, body self-concept, social self-concept
Externalizing	ADHD, attention, peer relationships, social functioning, extraversion and psychoticism, conduct and behavioral and hyperactivity subscales of Strength and Difficulties Questionnaire [34], behavioral problems
Language	Non-verbal communication skills, verbal memory, vocabulary, reading, spelling, letter–word identification, phonological processing

To reduce heterogeneity among studies for meta-analyses, we analysed studies using severe (birthweight < 1500 g and/or gestational age < 34 weeks) and moderate (birthweight < 2500 g and/or gestational age < 37 weeks) criteria separately. Where a study examined two categories of exposure that both fit our categorization of severe or moderate, we selected the exposure expected to be more debilitating, e.g., one study [35] categorized preterm (< 30 weeks) infants by whether they were intrauterine growth restricted (IUGR) or appropriate for gestational age (AGA) and so we only included the IUGR group. Another examined the effects of prematurity and small for gestational age (SGA) separately [36], and we included only the prematurity results, since these were more comparable with most other studies.

Meta-analysis were implemented in the R package ‘metafor’, using the ‘escalc’ function to calculate the standardized mean difference (SMD) for each study and sex, where SMD is the difference in mean value between affected and control children, divided by the pooled standard deviation of the two groups. We used the ‘rma.mv’ function to fit a random effects model, with sex and study as random effects [37]. The effect of exposure was estimated in males and females separately in each study, and then averaged over all studies, allowing us to test whether the effect of exposure differs by sex. Age at assessment was included as a moderator. Where subjects were within a range of ages upon assessment (and not measured at multiple discrete timepoints), the average age at assessment was used, if provided, and otherwise the midpoint of the range was used. Residual heterogeneity was assessed using the *Q*_E_ test, and the *I*^2^ statistic was calculated using the ‘rma’ function without random effects. Forest plots were used to visualize the results of individual studies.

For studies not included in the meta-analysis, because means and standard deviations could not be extracted, we performed a descriptive synthesis, summarizing traits where males were more affected, where females were more affected, where the sexes were affected equally and where there was no effect of exposure. We also considered whether results depended on how the outcome was assessed (e.g., researcher, parent, teacher or self), since bias in behavioural assessment may vary among these approaches.

### Reporting bias assessment and certainty assessment

Reporting bias assessment was performed by inspection of funnel plots. Since we did not seek to use specific estimates of effect to support clinical decisions or recommendations, we did not assess certainty of evidence [38].

## Results

### Study selection, study characteristics and results of individual studies

**Fig. 1:**
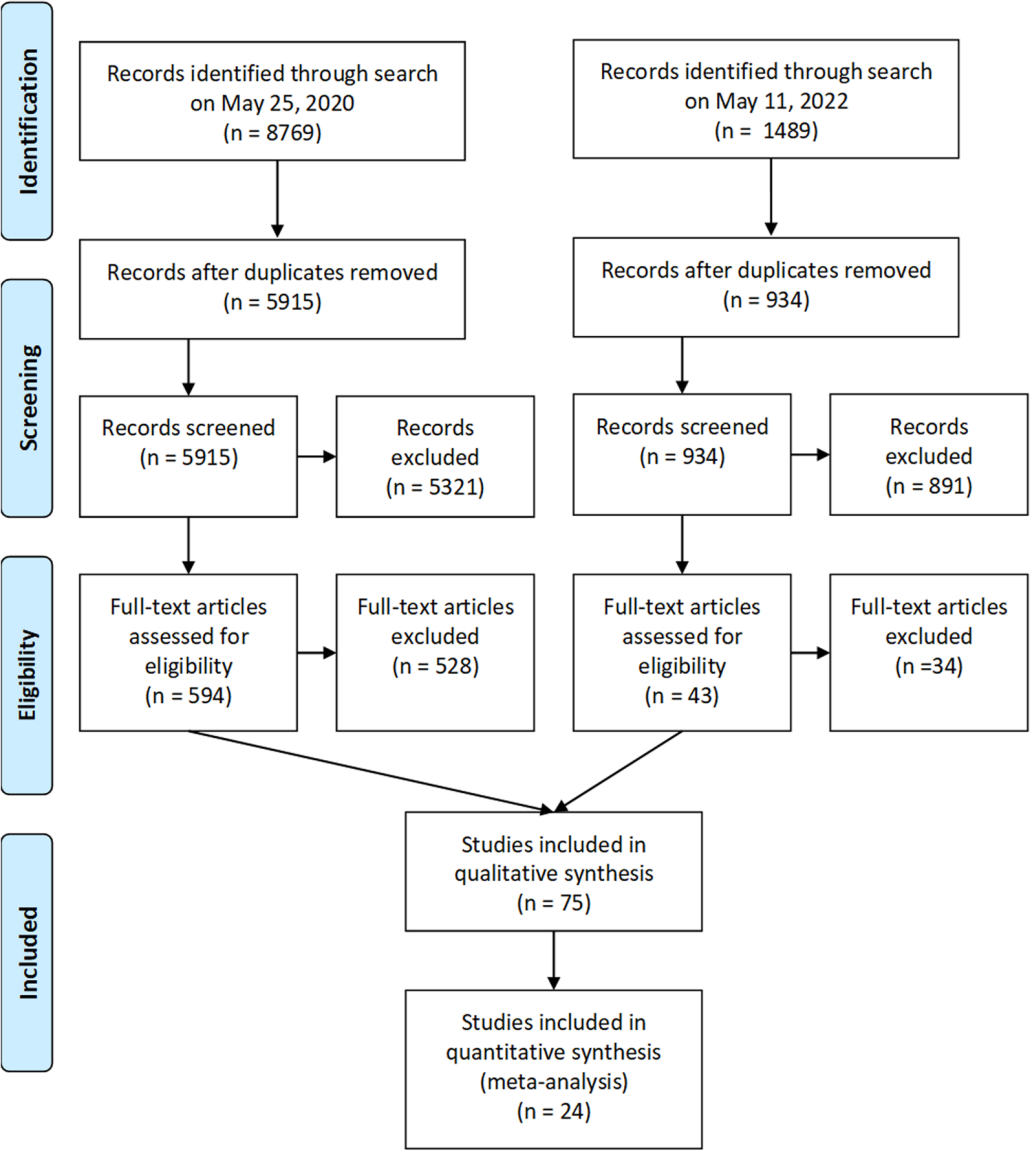
Flow diagram showing study selection. Reasons for exclusion at assessment of full-text articles included outcomes that were not of interest, results that not presented in a way that allowed assessment of sex dependence, or the absence of term/normal birthweight controls

Results of the search are shown in Fig. 1. In total, 75 studies matched our criteria [19, 35, 36, 39–110], although only 24 presented data in a way that could be extracted for meta-analyses [19, 35, 36, 41, 42, 51, 52, 61–66, 68, 73, 74, 76, 81, 85, 87, 89, 93, 105, 107]. Study characteristics and results of all studies are summarized in Additional file 2: Table S2, and the results categorized into cognitive function, internalizing behaviour, externalizing behaviour or language skills by age at assessment are provided in Additional file 2: Table S3. Data extracted from studies included in meta-analyses are provided in Additional file 2: Table S4.

### Risk of bias in studies

The results of assessment of quality and risk of bias for the 24 studies included in meta-analyses are shown in Additional file 2: Table S1. Given that we examined studies of the effects of preterm birth/low birthweight on neurodevelopmental outcomes, a number of the assessment criteria were necessarily true, whereas another assessment criterion was never met (please see Additional file 2: Table S1 for details). In other cases, our inclusion criteria ensured that the assessment criteria were met. With these caveats, the average score was 9.25 out of a possible 14 marks (66%) on the National Institutes of Health NHLBI Quality assessment tool for observational cohort and cross-sectional studies scale, which was considered intermediate quality [27]. The average score was 7.29 out of a possible score of 9 (81%) on the Newcastle–Ottawa scale, which was considered good [111]. A common weakness was that many studies used self- or parent-reported data, and so the assessment was not blind to exposure status. Even where an investigator or clinician was administering the test, it was generally not clear if they were blind to exposure status. Loss to follow up of over 20% was also common, occurring in over half of the studies.

### Descriptive synthesis

**Fig. 2:**
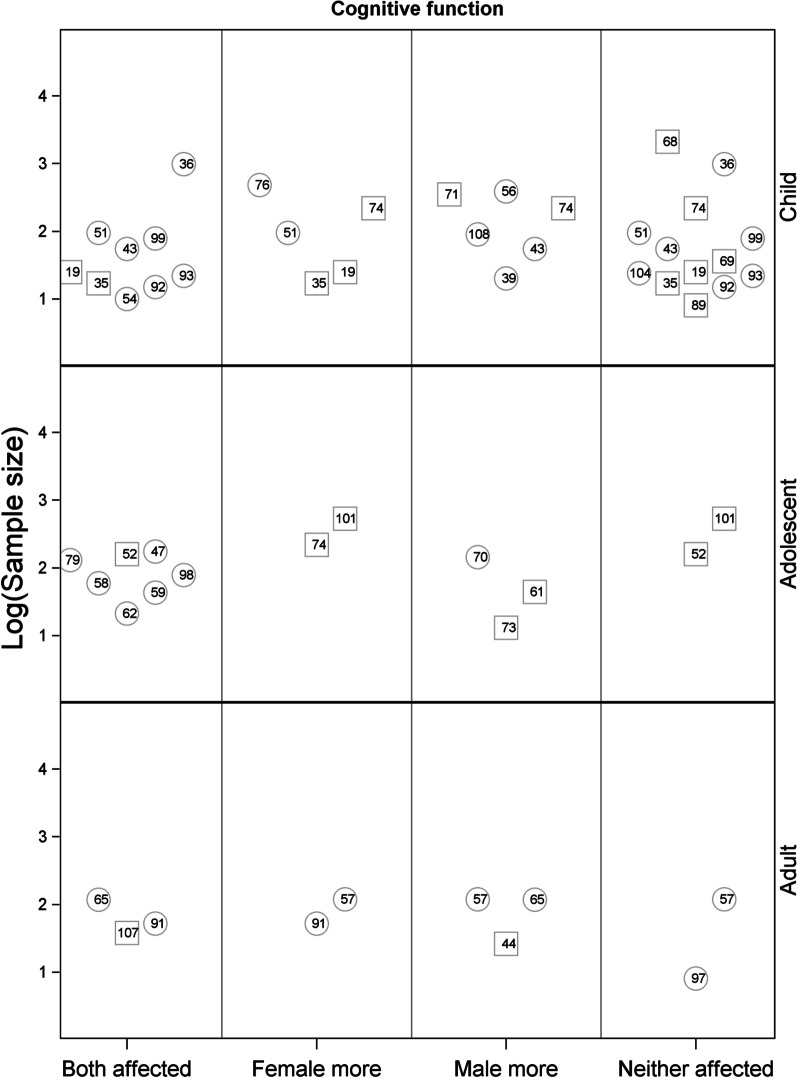
Qualitative synthesis of effects of prematurity/low birthweight on measures of cognitive function. Numbers indicate studies that found measures of cognitive function to be affected in both sexes, to be affected more in females, to be affected more in males, or to be affected in neither sex in childhood (1–10 years), adolescence (11–18 years), and adulthood (over 19 years). In each age group, the *y*-axis indicates the logarithm (base 10) of the sample size. A single study could show different results for different traits and so may appear in more than one cell. Study numbering is the same as in text. Circles indicate studies that examined sex-dependence using statistical interaction terms, whereas squares indicate studies that analyzed the sexes separately

**Fig. 3:**
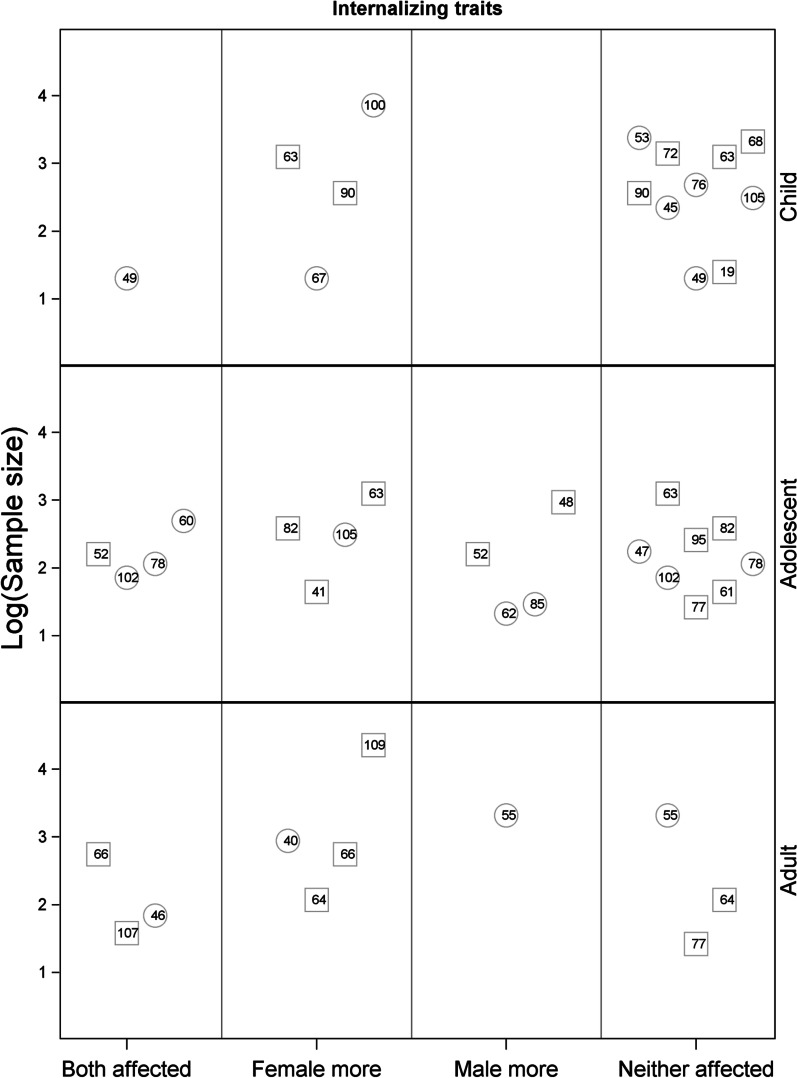
Qualitative synthesis of effects of prematurity/low birthweight on internalizing traits

**Fig. 4:**
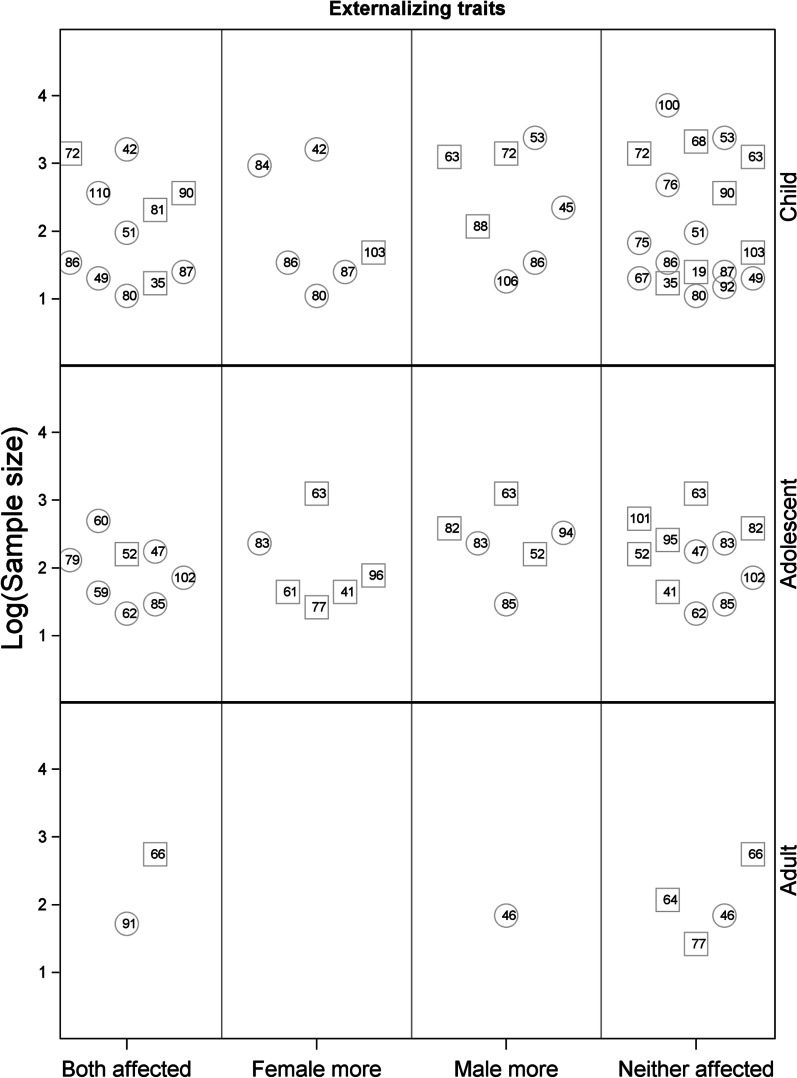
Qualitative synthesis of effects of prematurity/low birthweight on externalizing traits

**Fig. 5:**
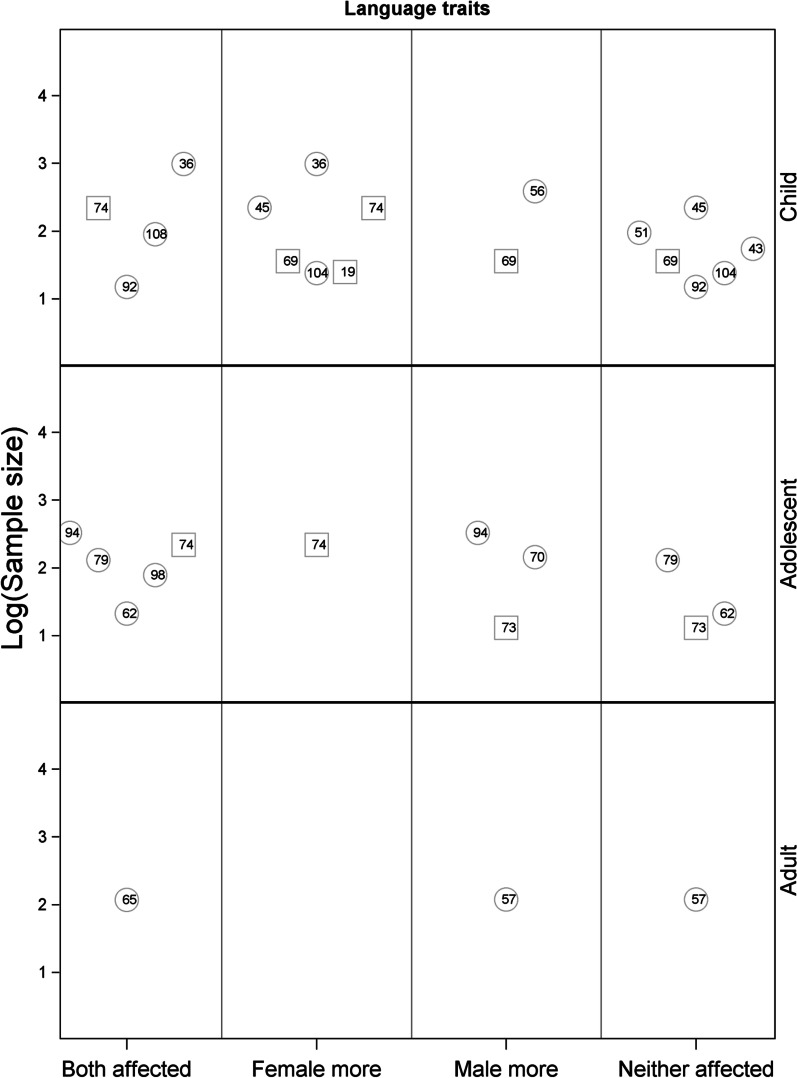
Qualitative synthesis of effects of prematurity/low birthweight on language traits

Figures 2, 3, 4 and 5 summarize whether effects of prematurity/low birthweight were sex-dependent, significant in both sexes, or not significant in either sex, for traits related to cognitive function, internalizing behaviour, externalizing behaviour or language skills, respectively; trait categories are described in Table 1. Only 4 studies [45, 50, 53, 60] reported autism-related traits, and so these were not included in figures. Overall, sex-dependent effects tended to be less frequent than findings of effects in both sexes or findings of no effect in either sex. In general, there was no obvious excess or deficiency of male-biased or female-biased effects for any trait category. However, four studies [63, 67, 90, 100] found internalizing and emotional problems to be more affected in females in childhood (10 years of age or lower), whereas no study found males to be more affected at this age (Fig. 3). However, two of these studies [63, 90] observed other internalizing traits to be unaffected in either sex. Language traits in childhood also were also more often affected in females [19, 36, 45, 69, 74, 104] than in males [56, 69] (Fig. 5). However, all but one of the studies that found traits more affected in females [19] also found other traits to be affected in both sexes or unaffected in either sex.

Studies that did not find an effect of prematurity/low birthweight did not have obviously lower sample sizes than those reporting significant effects (Figs. 2, 3, 4 and 5). For most types of traits, more studies assessed traits in childhood, although for internalizing traits, there were similar number of assessments at adolescence.

Testing whether effects are sex-dependent by testing the sexes separately, rather than a more formal test (e.g., of the interaction between sex and exposure) increases the frequency of false positives [112, 113]. We therefore expected that studies testing the sexes separately would show more sex-dependent effects than studies that tested for interactions between sex and exposure. However, this was not observed (Figs. 2, 3, 4 and 5).

### Effects of observer

A few studies had traits assessed by both youth and parents, or by parents and teachers, allowing the effects of different observers to be compared directly. The results of self-reports often differed from those of parent/care-giver reports, although not in consistent ways. For internalizing problems, two studies found sex-dependent effects of prematurity/low birthweight in self reports but not parent reports [52, 66], whereas this pattern was reversed in a third [64]. For externalizing problems, effects of prematurity/low birthweight in both sexes were observed in self reports but in neither sex in parent reports [52], whereas another study found the reverse [66], and a third study found no effects in either self reports or parent reports [64]. Taylor et al. found sex by observer interactions for a variety of internalizing and externalizing traits that indicated that differences between parent and self reports were larger for females than for males [102].

Parent and teacher reports both found effects on autism and ADHD symptoms in both sexes [60]. However, in another study, teacher-reported disattention showed a greater effect in females, and teacher-reported hyperactivity/impulsivity was not affected in either sex, whereas both of these traits were affected in both sexes when reported by parents [87]. Thus, the observer may be a source of heterogeneity in such studies, although its effects do not appear to be consistent.

### Quantitative synthesis—severe prematurity/low birthweight on cognitive function

**Fig. 6:**
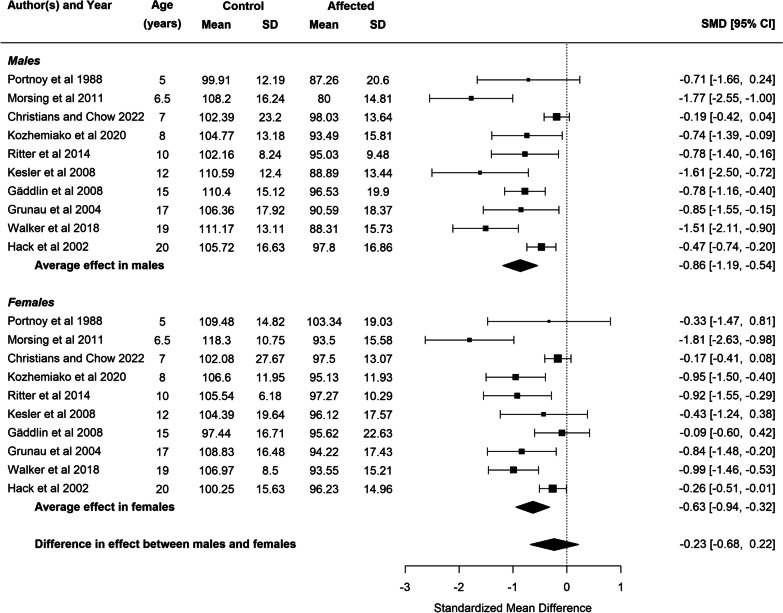
Meta-analysis of the effects of severe prematurity/low birthweight on cognitive function. Squares represent estimates (with confidence intervals) and marker size indicates weight. Diamonds represent estimates for each sex and for the difference between sexes, with the width of the diamond indicating the confidence interval. *SMD* standardized mean difference

Ten studies examined the effects of severe prematurity/low birthweight on cognitive function, generally measured as IQ [19, 35, 36, 61, 62, 65, 73, 89, 93, 107]. Age was not a significant moderator (*P* = 0.80; Additional file 3: Fig. S1) and so was removed from the model. Severe prematurity/low birthweight significantly reduced cognitive function (Fig. 6), but this effect did not differ between males and females (*P* = 0.31). There was significant, high heterogeneity among studies (*I*^2^ = 76%, *Q*_E_ = 68, *P* < 0.0001), although the overall result is generally consistent with the results of individual studies, most of which found both sexes to be affected [19, 35, 36, 62, 65, 93, 107]. However, two studies found a significant effect in males but not females [61, 73], while another found no effect in either sex [89].

### Quantitative synthesis—moderate prematurity/low birthweight on cognitive function

**Fig. 7:**
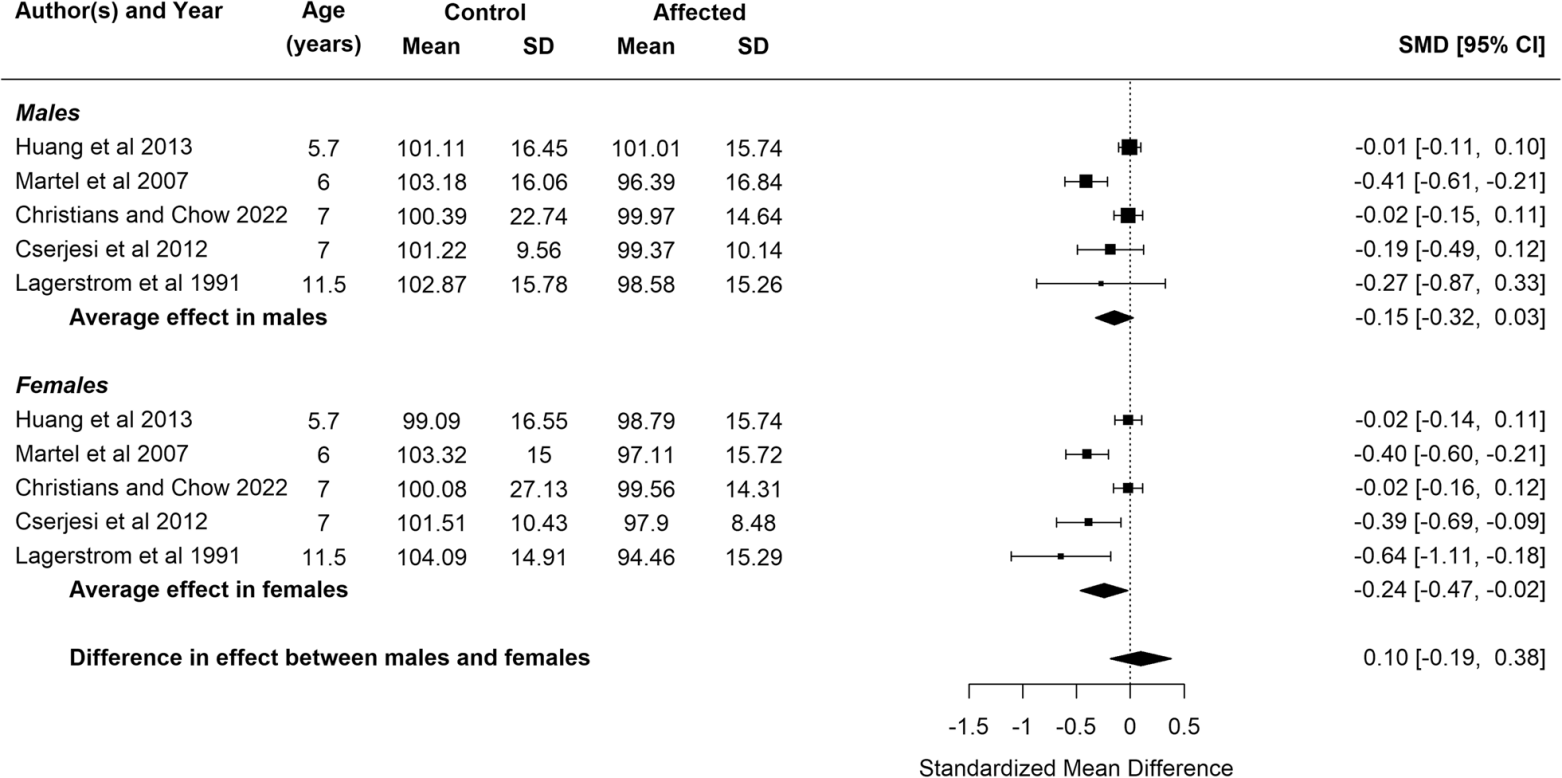
Meta-analysis of the effects of moderate prematurity/low birthweight on cognitive function

Five studies examined the effects of moderate prematurity/low birthweight on cognitive function [36, 51, 68, 74, 81], and age was not a significant moderator (*P* = 0.24; Additional file 3: Fig. S2). Moderate prematurity/low birthweight reduced cognitive function (Fig. 7), but males and females did not differ in estimated effect size (*P* = 0.50). There was significant heterogeneity among studies (*I*^2^ = 80%, *Q*_E_ = 35, *P* < 0.0001). The two individual studies with sample sizes greater than 200 in all groups found no effect [36, 68], while another found an effect in both sexes [81], and two others found a variety of patterns depending on which aspect of cognitive function was examined [51, 74].

### Quantitative synthesis—severe prematurity/low birthweight on internalizing problems

**Fig. 8:**
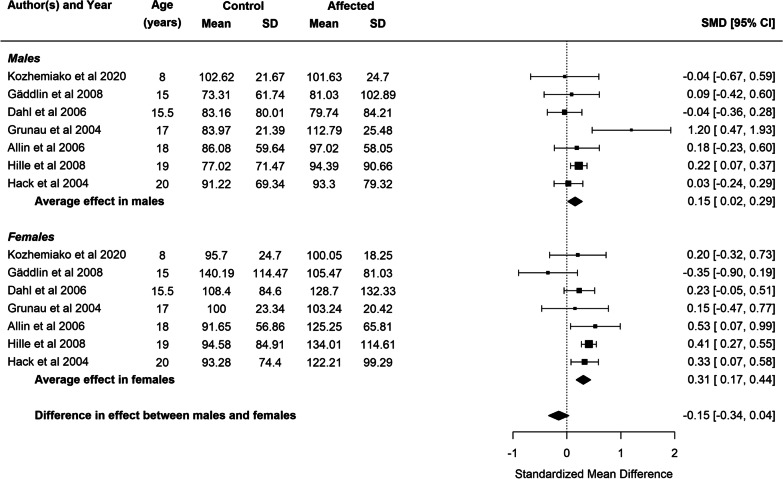
Meta-analysis of the effects of severe prematurity/low birthweight on internalizing problems

Seven studies examined the effects of severe prematurity/low birthweight on internalizing problem scores [19, 41, 52, 61, 62, 64, 66]. Age was not a significant moderator (*P* = 0.11; Additional file 3: Fig. S3) and so was removed from the model. Severe prematurity/low birthweight significantly increased internalizing problem scores (Fig. 8), and this effect tended to be larger in females, but the difference between the sexes was not significant (*P* = 0.12). The heterogeneity among studies was marginally non-significant (*I*^2^ = 19%, *Q*_E_ = 20, *P* = 0.06). Within individual studies, results varied among different internalizing traits or were sometimes sex-dependent [41, 52, 62, 64, 66], although two individual studies found no significant effects [19, 61]. Only four studies examined the effects of moderate prematurity/low birthweight on internalizing problems [63, 68, 81, 105], and so these results were not synthesized.

### Quantitative synthesis—severe and moderate prematurity/low birthweight on externalizing problems

**Fig. 9:**
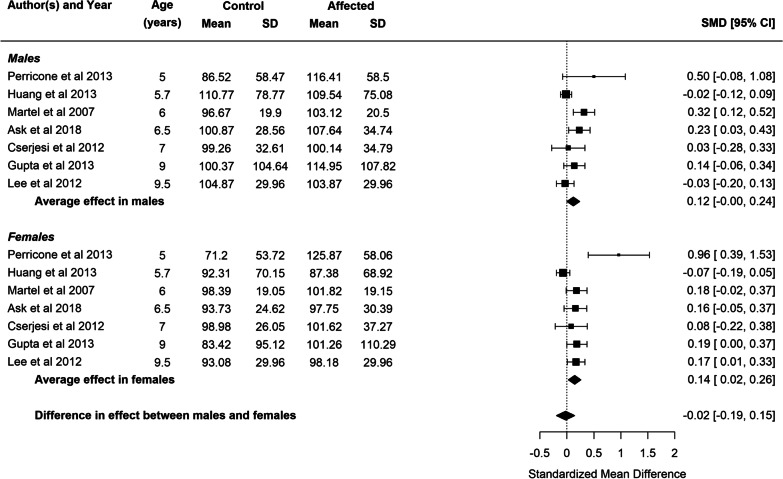
Meta-analysis of the effects of moderate prematurity/low birthweight on externalizing problems

**Fig. 10:**
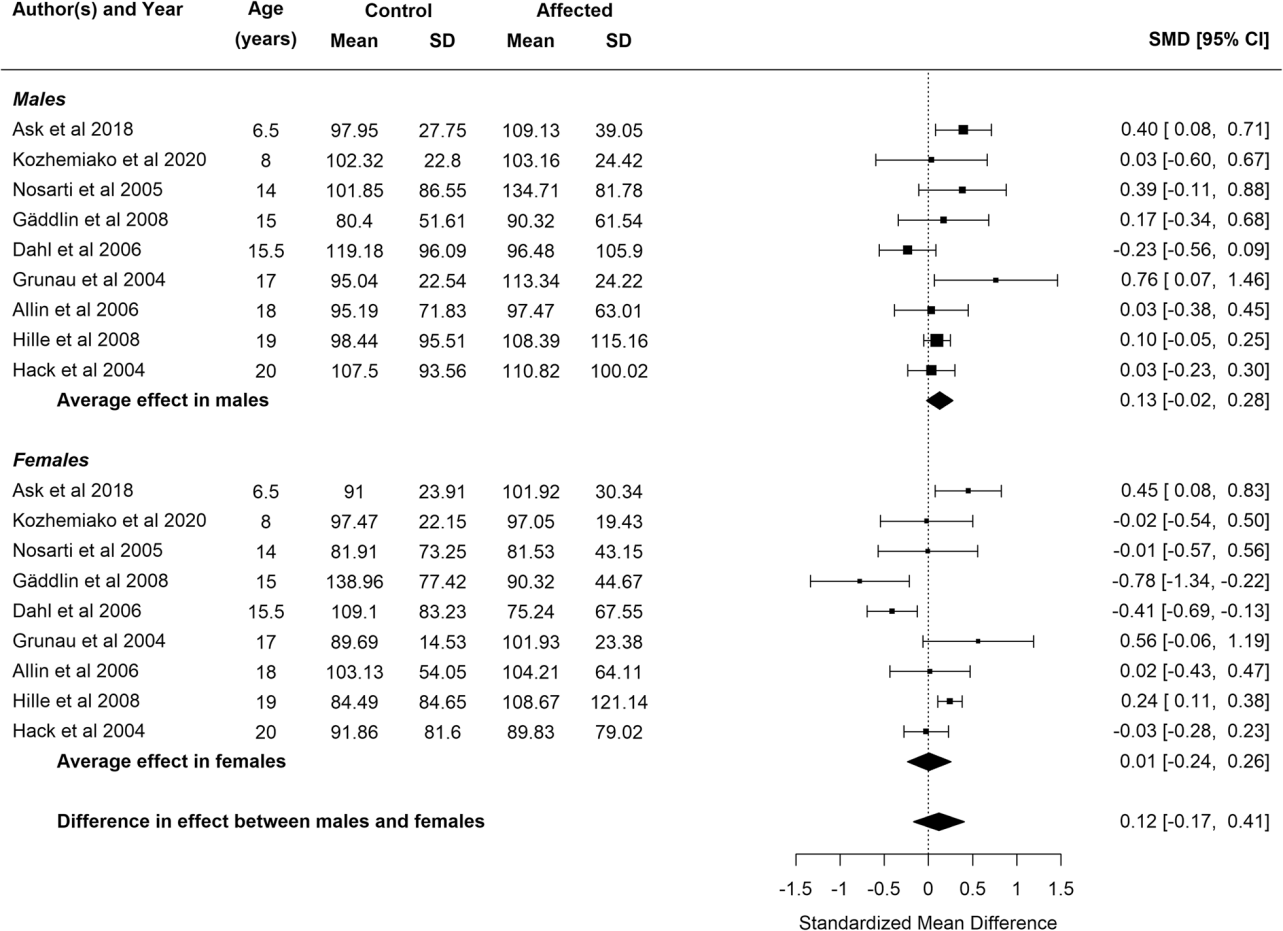
Meta-analysis of the effects of severe prematurity/low birthweight on externalizing problems

Nine and 7 studies examined the effects of severe [19, 41, 42, 52, 61, 62, 64, 66, 85] and moderate [42, 51, 63, 68, 76, 81, 87] prematurity/low birthweight on externalizing problem scores, respectively. Age was not a significant moderator in either analysis (*P* = 0.17 and 0.42, respectively; Additional file 3: Figs. S4 and S5) and so was removed from the models. Surprisingly, moderate prematurity/low birthweight significantly increased externalizing problem scores (Fig. 9), whereas severe prematurity/low birthweight did not (Fig. 10). However, in neither case did effect sizes differ between the sexes (*P* = 0.42 and 0.78 for severe and moderate, respectively). There was significant heterogeneity among studies (*I*^2^ = 67%, *Q*_E_ = 46, *P* < 0.0001, and *I*^2^ = 59%, *Q*_E_ = 34, *P* = 0.0007 for severe and moderate, respectively). Among studies examining effects of severe prematurity/low birthweight on externalizing traits, three individual studies found no effects [19, 41, 64], while others found greater effects in females [61] or effects in both sexes [62], or variable effects in different traits [42, 52, 66, 85]. Among studies examining effects of moderate prematurity/low birthweight, most reported variable patterns among different externalizing traits [42, 51, 63, 87], although two, including one with sample sizes greater than 200 in all groups, found no significant effects [68, 76].

### Quantitative synthesis by observer

Because we found that observer (e.g., self vs parent) may be a source of heterogeneity, we repeated meta-analyses of internalizing and externalizing traits separately for self-reports and parent-reports. In no case did the effect of prematurity/low birth weight differ between males and females (Additional file 4). However, for the effects of severe prematurity/low birthweight on both internalizing problem scores and externalizing problem scores, heterogeneity was higher for self-reports (*I*^2^ = 76% and 85% for internalizing and externalizing, respectively) than for parent reports (0% and 46%; Additional file 4). Severe prematurity/low birthweight significantly increased parent-reported internalizing problem scores, as in the combined analysis (described above), but did not have a significant effect on self-reported internalizing problem scores (Additional file 4). Severe prematurity/low birthweight did not have significant effects on self-reported or parent-reported externalizing problem scores (Additional file 4), as in the combined analysis. The effect of moderate prematurity/low birthweight on parent-reported externalizing problem scores was marginally non-significant (*P* = 0.06; Additional file 4), whereas it was significant in the combined analysis. Heterogeneity was similar in the parent-reported studies (*I*^2^ = 61%; Additional file 4) as in the combined analysis (*I*^2^ = 59%). There were no studies of the effects of moderate prematurity/low birthweight on self-reported externalizing problem scores.

### Reporting biases

Funnel plots did not show asymmetry (Additional file 3: Figs. S6–S10), suggesting no evidence of reporting bias.

## Discussion

This is the first systematic review and meta-analysis to examine whether the effects of prematurity/low birthweight on neurodevelopmental outcomes are sex-dependent, and one of the first to examine sex-dependent long-term effects of prenatal adversity in humans (e.g., [27]). In our quantitative synthesis, we found no significant sex-dependence of effects of severe or moderate prematurity/low birthweight on cognitive function, internalizing traits or externalizing traits. Severe prematurity/low birthweight tended to have greater effects on internalizing problem scores in females, but this was not significant and effect sizes were small, i.e., a SMD of 0.15 in males and 0.31 in females. In most meta-analyses, heterogeneity was significant and moderate (*I*^2^ > 50%) to high (*I*^2^ > 75%). We used a random effects model, which accounts for variability between studies [114], but nevertheless it appears that the effects of prematurity/low birthweight on cognitive function, internalizing problems and externalizing problems are not consistent. While this precludes a definitive conclusion on the overall effects of prematurity/low birthweight, we can conclude with confidence that one sex is not consistently more affected than the other.

To assess potential sources of heterogeneity, we analysed effects of severe and moderate prematurity/low birthweight separately, and included age at assessment as a moderator, which was not found to be significant. Recently, it was found that the type of test used to assess cognitive abilities (e.g., full-scale vs short-form assessments of general intelligence) contributed to 14% of between-study variance in the effects of prematurity [115]. In the present study, this may have contributed to the heterogeneity between studies, although a number of studies were ambiguous about whether the assessment was full-scale or short-form, and so we could not assess this formally. We also examined studies which reported traits assessed by different observers (i.e., youth and parents or parents and teachers) and found results often differed depending on observer, but not in a consistent way, although one study found that differences between parent and self reports were larger for females than for males [102]. Gendered expectations of behaviour may also contribute to variability in assessments [76], and this effect may depend on specific social context.

In our descriptive synthesis of 75 studies, we found that sex-dependent effects tended to be less frequent than findings of effects in both sexes or neither sex, and generally there did not appear to be an excess or deficiency of male-biased or female-biased effects. There were slight excesses of results finding internalizing and language problems to be more affected in females in childhood. However, even in these categories, there were many more results of effects in both sexes and/or neither sex, even among studies with substantial sample sizes. This is consistent with the results of the meta-analyses, which found no overall sex dependence.

Numerous studies in this field examine sex differences in responses to early life adversity without explicitly testing whether effects differ between males and females (e.g., using an interaction) [20, 60]. Testing males and females separately would be expected to generate spurious sex-dependent effects [36, 112, 113]. In the present study, approximately two-thirds of included studies tested interactions between prematurity/low birthweight and sex, while the remainder of the studies tested effects in males and females separately. Surprisingly, studies that tested the sexes separately did not show an excess of sex-dependent effects.

## Limitations

Definitions of prematurity/low birthweight varied among studies, which may explain some of the heterogeneity among studies, although a previous meta-analysis has supported the use of both gestational age and/or birthweight as inclusion criteria [30]. The wide variety of tools used to assess outcomes likely contributed to heterogeneity as well. The lack of consistency in how data were analysed and how results were reported made synthesis challenging. While our results show that one sex is not consistently more affected than the other, it is possible that there are combinations of exposure severity, outcome, age and mode of assessment, postnatal environment, etc., where one sex is consistently more vulnerable. However, the lack of identifiable causes of heterogeneity, resulting from variability in both study design and reporting [115], means that we are currently unable to identify consistent sex-dependent effects, if they occur. Variable effects of gender in different populations may have also contributed to heterogeneity. While we used the term “sex” for brevity and because the exposure occurred prior to birth, we acknowledge that outcomes may have been heavily influenced by gender; we did not identify any studies that sought to disentangle effects of sex and gender. Gendered treatment of children may have diminished or enhanced biologically based sex differences in the effects of prematurity/low birthweight, e.g., where one gender receives more support in the development of certain traits and behaviours, or is subject to more rigid social expectations, observed effects of adverse early life conditions may be reduced in that gender.

Our conclusions are also limited by the underlying studies. Many studies used self- or parent-reported data and/or it was not clear if the assessor was blind to exposure status. Combined with gendered expectations, this lack of blinding may have obscured or exaggerated sex-dependent effects. Loss to follow up of over 20% was also common, occurring in over half of the studies. However, it is not clear whether bias introduced by loss to follow up would affect the sexes and genders differently. Moreover, a recent meta-regression found that attrition rate did not contribute to variation in effect sizes in studies of preterm birth and cognitive ability [115].

A difficulty in interpreting sex-dependent effects of early life adversity is that the prevalence and severity of adversity may differ between the sexes. Indeed, males are at increased risk of preterm birth [116, 117]. This may create a selection bias, e.g., if male newborns are more likely to die and thus be lost to follow-up. This might dampen sex-dependent effects if, for example, males were more impacted by prematurity, but the most-severely affected males did not survive, whereas females did. However, such effects of selection would be expected to be reduced in cases of moderate prematurity/low birthweight, because a greater proportion of infants would be expected to survive. We did not observe greater sex-dependence in our analysis of moderate complications. The issue of selection bias is also difficult to address, because mortality is female-biased early in gestation [118], i.e., while there may be an observable bias, where males are more likely to be lost to follow-up, there may also be an unobserved bias, where females are more likely to be lost at earlier stages of gestation and so not included in studies at all.

## Perspectives and significance

It has often been suggested that males have greater susceptibility to early life conditions [15–23]. Our results show that this is not the case with regard to the lasting effects of premature birth and/or low birthweight on cognitive function, internalizing problems and externalizing problems. Thus, the view that males are more vulnerable in general should be re-evaluated. Specific insults may have sex-dependent effects on specific phenotypes [27], but care should be taken in generalizing such observations. While sex and gender clearly influence health and disease, as well as the effects of early life adversity, it is also important to acknowledge that many traits may not show such differences.

## Supplementary Information

**Additional file 1.** Search strategy.

**Additional file 2.** Additional Tables S1–S4.

**Additional file 3: Figure S1.** Effect of age as a moderator on the effect of severe prematurity/low birthweight on cognitive function. Squares and circles are estimates for males and females, respectively, and marker size indicates weight. **Figure S2.** Effect of age as a moderator on the effect of moderate prematurity/low birthweight on cognitive function. **Figure S3.** Effect of age as a moderator on the effect of severe prematurity/low birthweight on internalizing problems. **Figure S4.** Effect of age as a moderator on the effect of severe prematurity/low birthweight on externalizing problems. **Figure S5.** Effect of age as a moderator on the effect of moderate prematurity/low birthweight on externalizing problems. **Figure S6.** Funnel plot of residuals (observed–fitted values) and standard errors for the effect of severe prematurity/low birthweight on cognitive function. Squares and circles are estimates for males and females, respectively, and marker size indicates weight. **Figure S7.** Funnel plot for the effect of moderate prematurity/low birthweight on cognitive function. **Figure S8.** Funnel plot for the effect of severe prematurity/low birthweight on internalizing problems. **Figure S9.** Funnel plot for the effect of severe prematurity/low birthweight on externalizing problems. **Figure S10.** Funnel plot for the effect of moderate prematurity/low birthweight on externalizing problems.

**Additional file 4.** Additional results.

## Data Availability

This review was based on published data. All data extracted for this study are included in the article and its Additional files.
