# Supplementary material for: Sex differences in the effects of prematurity and/or low birthweight on neurodevelopmental outcomes: systematic review and meta-analyses
Source: Biol Sex Differ. 2023 Jul 11;14:47. doi: 10.1186/s13293-023-00532-9 (PMC10334669; doi:10.1186/s13293-023-00532-9)
Supplement: Supplementary file 1 — Additional file 1. Search strategy. [file 13293_2023_532_MOESM1_ESM.pdf]

For all database searches, we combined the "Sex difference", "Prenatally programmed", and Neurodevelopment" results (using AND terms), and excluded "Animal studies" and "Toxicological studies" results (using NOT terms).

**Web of Science**	
**Concept**	**Keywords**
Sex difference	TS =((sex NEAR/2 difference*) OR (gender NEAR/2 difference*) OR (sex NEAR/2 characteristic*) OR (gender NEAR/2 characteristic*) OR (sex NEAR/4 specific) OR (gender NEAR/4 specific) OR (sex NEAR/4 dependent) OR (gender NEAR/4 dependent) OR (sexual* NEAR/2 dimorph*) OR (moderated NEAR/4 sex))
Prenatally programmed	TS = (prenatal* OR "in utero" OR fetal OR foetal OR preterm OR prematur* OR birthweight OR (birth NEAR/2 weight) OR (prenatal maternal NEAR/2 (stress OR depression OR anxiety)))
Neurodevelopment	TS = (neurodevelopment OR cogniti* OR neurologic* OR academic OR education* OR executive OR attention OR learning OR memory OR language OR intelligen* OR aptitude OR (mental NEAR/2 health) OR emotion* OR behavior* OR behaviour*)
Exclude animal studies	TS = ((rodent OR mouse OR mice OR rat OR rats) NOT ("human" OR "humans" OR child OR baby))
Exclude toxicological studies	TS = (pollut* OR contamin* OR toxicity OR "heavy metal" OR pesticide OR "bisphenol A")

**Scopus**	
**Concept**	**Keywords**
Sex difference	TITLE-ABS-KEY((sex W/2 difference*) OR (gender W/2 difference*) OR (sex W/2 characteristic*) OR (gender W/2 characteristic*) OR (sex W/4 specific) OR (gender W/4 specific) OR (sex W/4 dependent) OR (gender W/4 dependent) OR (sexual* W/2 dimorph*) OR (moderated W/4 sex))
Prenatally programmed	TITLE-ABS-KEY (prenatal* OR "in utero" OR fetal OR foetal OR preterm OR prematur* OR birthweight OR (birth W/2 weight) OR (prenatal maternal W/2 (stress OR depression OR anxiety)))
Neurodevelopment	TITLE-ABS-KEY (neurodevelopment OR cogniti* OR neurologic* OR academic OR education* OR executive OR attention OR learning OR memory OR language OR intelligen* OR aptitude OR (mental W/2 health) OR emotion* OR behavior* OR behaviour*)
Exclude animal studies	TITLE-ABS-KEY ((rodent OR mouse OR mice OR rat OR rats) AND NOT ("human" OR "humans" OR child OR baby))
Exclude toxicological studies	TITLE-ABS-KEY (pollut* OR contamin* OR toxicity OR "heavy metal" OR pesticide OR "bisphenol A")

**Ovid Medline**	
**Concept**	**Keywords**
Sex difference	((sex ADJ2 difference*) OR (gender ADJ2 difference*) OR (sex ADJ2 characteristic*) OR (gender ADJ2 characteristic*) OR (sex ADJ4 specific) OR (gender ADJ4 specific) OR (sex ADJ4 dependent) OR (gender ADJ4 dependent) OR (sexual* ADJ2 dimorph*) OR (moderated ADJ4 sex))
Prenatally programmed	(prenatal* OR "in utero" OR fetal OR foetal OR preterm OR prematur* OR birthweight OR (birth ADJ2 weight) OR (prenatal maternal ADJ2 (stress OR depression OR anxiety)))
Neurodevelopment	(neurodevelopment OR cogniti* OR neurologic* OR academic OR education* OR executive OR attention OR learning OR memory OR language OR intelligen* OR aptitude OR (mental ADJ2 health) OR emotion* OR behavior* OR behaviour*)
Exclude animal studies	((rodent OR mouse OR mice OR rat OR rats) NOT ("human" OR "humans" OR child OR baby))
Exclude toxicological studies	(pollut* OR contamin* OR toxicity OR "heavy metal" OR pesticide OR "bisphenol A")
