# Supplementary material for: Sex differences in the effects of prematurity and/or low birthweight on neurodevelopmental outcomes: systematic review and meta-analyses
Source: Biol Sex Differ. 2023 Jul 11;14:47. doi: 10.1186/s13293-023-00532-9 (PMC10334669; doi:10.1186/s13293-023-00532-9)
Supplement: Supplementary file 3 — Additional file 3: Figure S1. Effect of age as a moderator on the effect of severe prematurity/low birthweight on cognitive function. Squares and circles are estimates for males and females, respectively, and marker size indicates weight. Figure S2. Effect of age as a moderator on the effect of moderate prematurity/low birthweight on cognitive function. Figure S3. Effect of age as a moderator on the effect of severe prematurity/low birthweight on internalizing problems. Figure S4. Effect of age as a moderator on the effect of severe prematurity/low birthweight on externalizing problems. Figure S5. Effect of age as a moderator on the effect of moderate prematurity/low birthweight on externalizing problems. Figure S6. Funnel plot of residuals (observed–fitted values) and standard errors for the effect of severe prematurity/low birthweight on cognitive function. Squares and circles are estimates for males and females, respectively, and marker size indicates weight. Figure S7. Funnel plot for the effect of moderate prematurity/low birthweight on cognitive function. Figure S8. Funnel plot for the effect of severe prematurity/low birthweight on internalizing problems. Figure S9. Funnel plot for the effect of severe prematurity/low birthweight on externalizing problems. Figure S10. Funnel plot for the effect of moderate prematurity/low birthweight on externalizing problems. [file 13293_2023_532_MOESM3_ESM.docx]

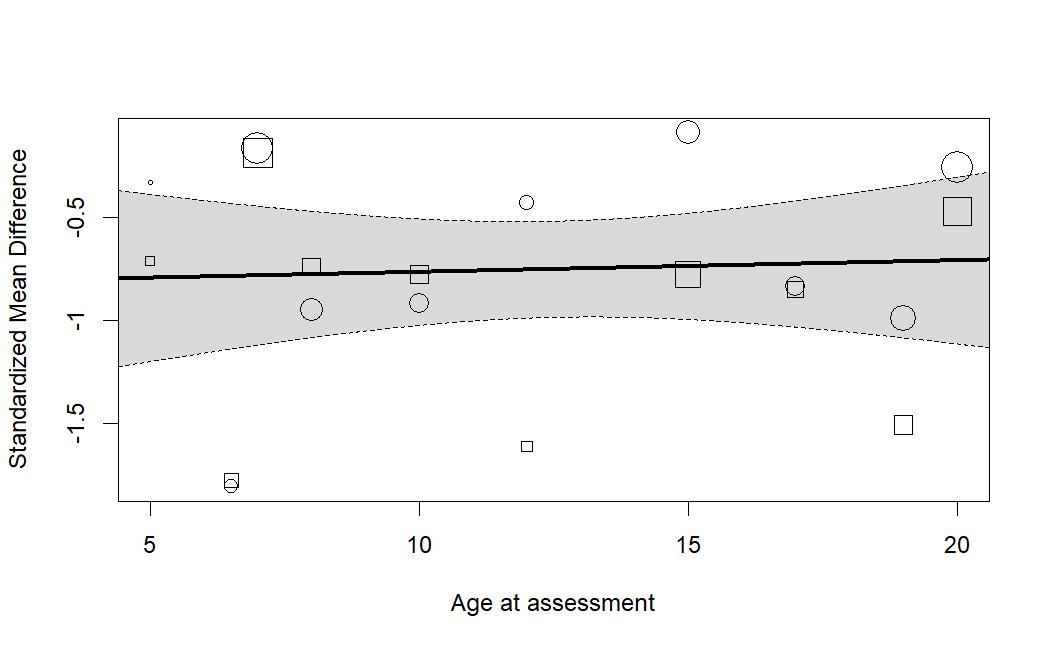


Figure S1. Effect of age as a moderator on the effect of severe prematurity/ low birthweight on cognitive function. Squares and circles are estimates for males and females, respectively, and marker size indicates weight.


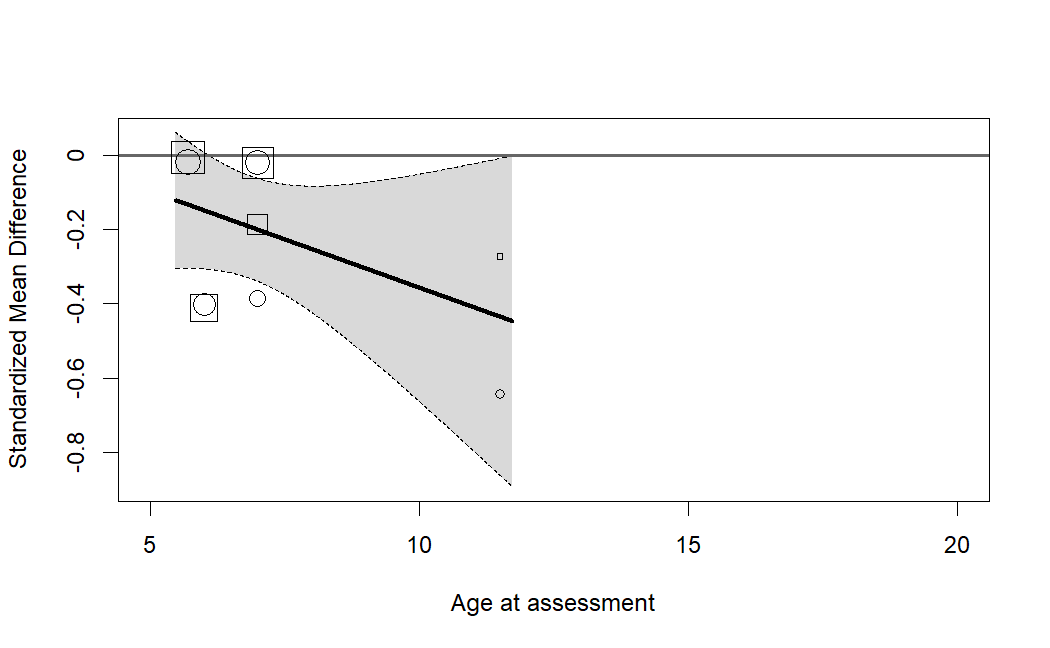


Figure S2. Effect of age as a moderator on the effect of moderate prematurity/ low birthweight on cognitive function.


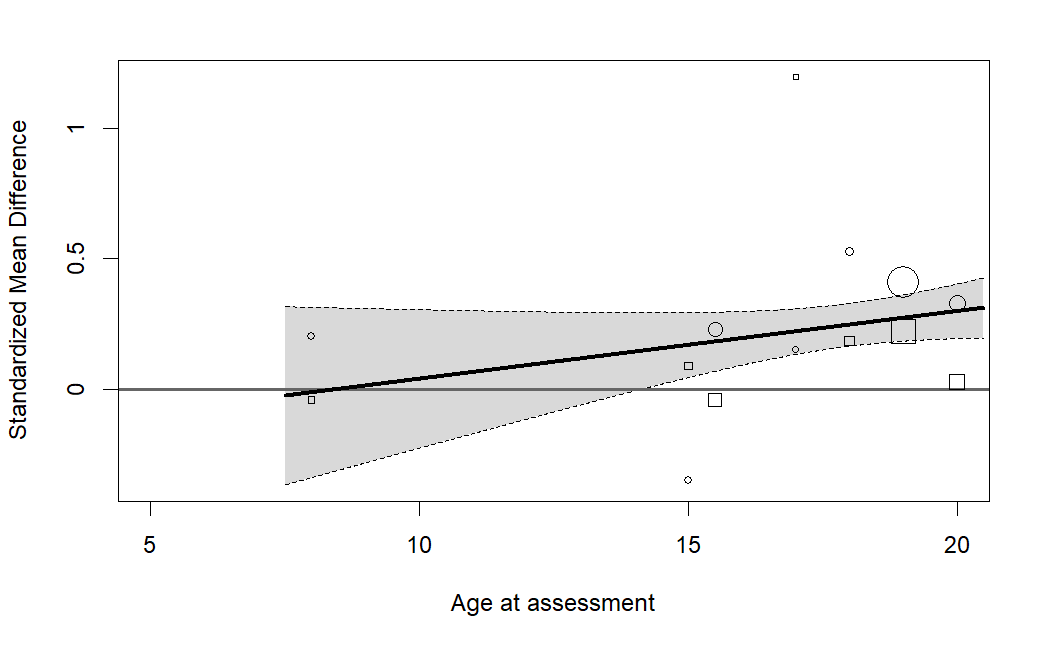


Figure S3. Effect of age as a moderator on the effect of severe prematurity/ low birthweight on internalizing problems.


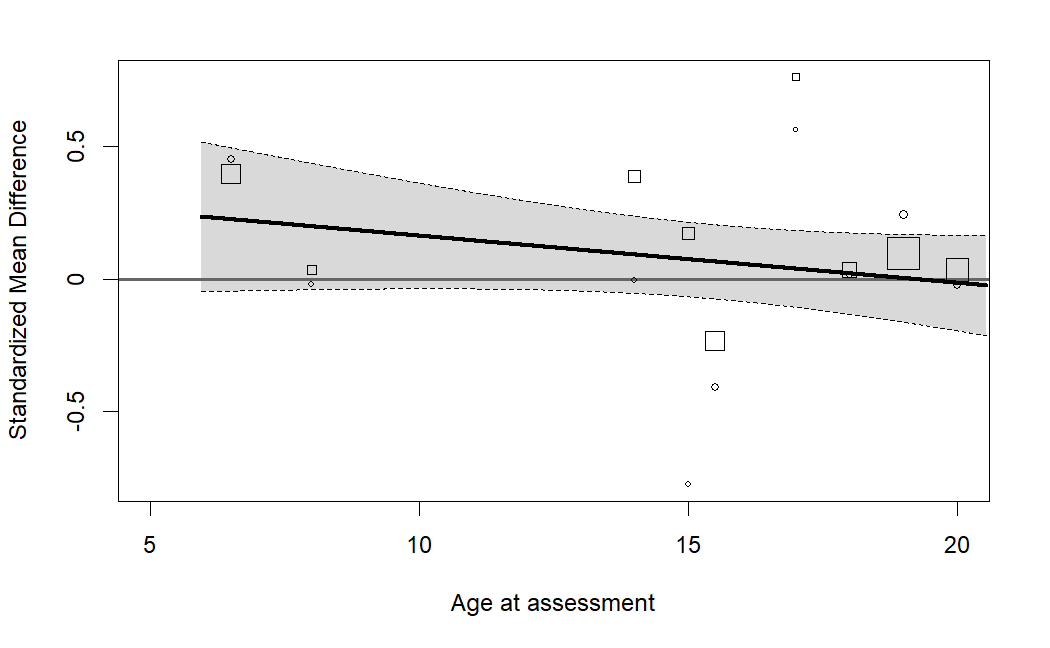


Figure S4. Effect of age as a moderator on the effect of severe prematurity/ low birthweight on externalizing problems.


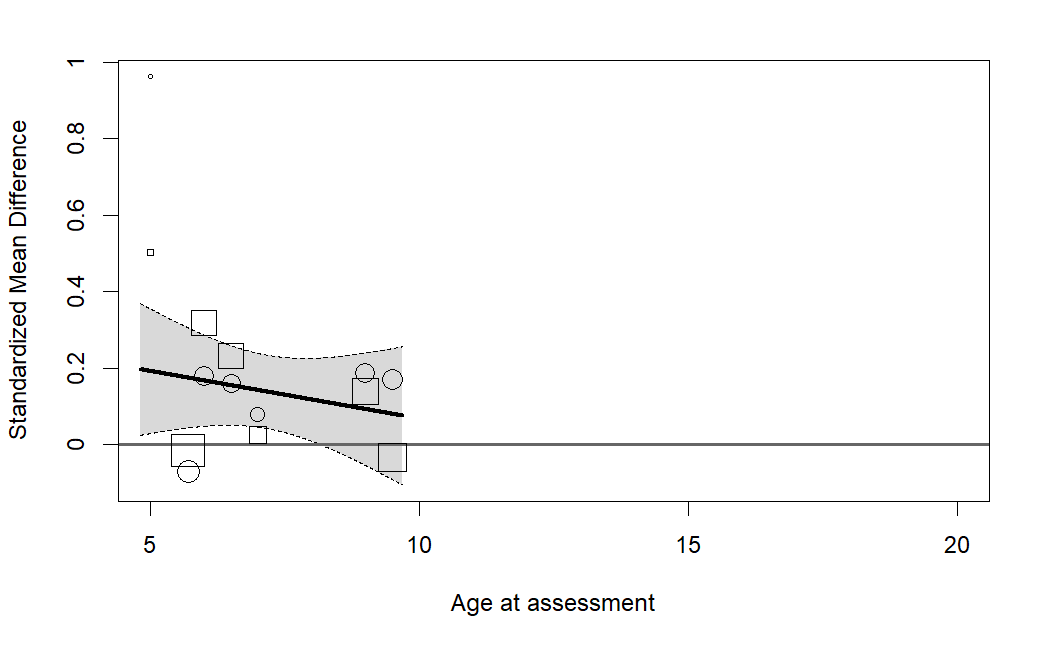


Figure S5. Effect of age as a moderator on the effect of moderate prematurity/ low birthweight on externalizing problems.


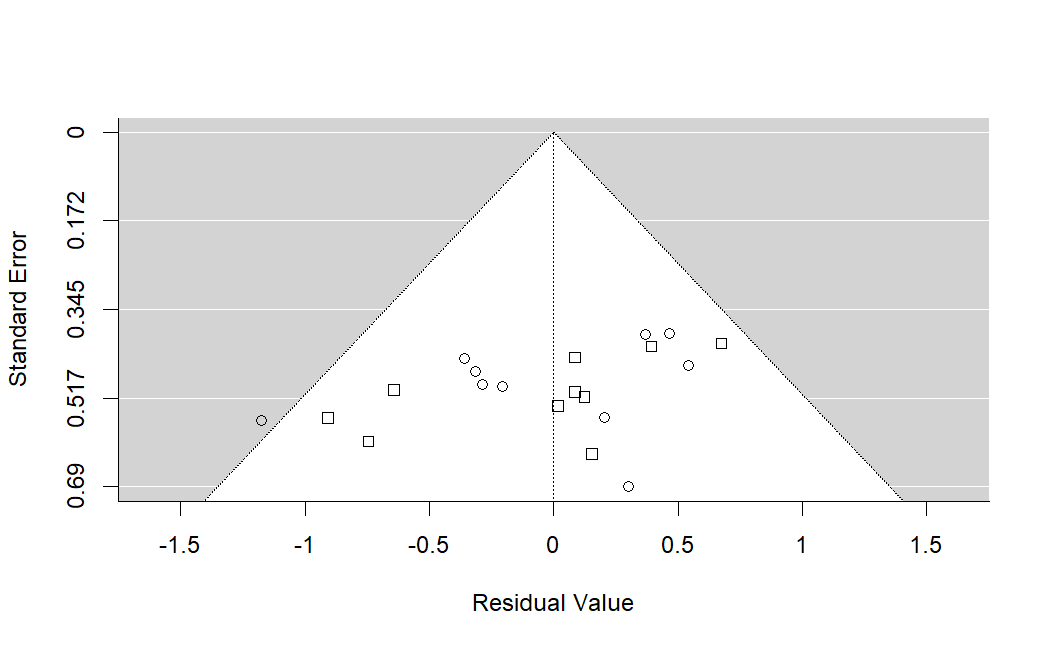


Figure S6. Funnel plot of residuals (observed – fitted values) and standard errors for the effect of severe prematurity/ low birthweight on cognitive function. Squares and circles are estimates for males and females, respectively, and marker size indicates weight.


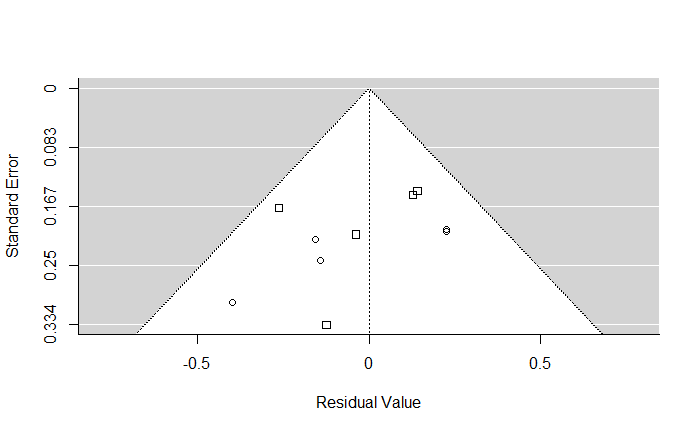


Figure S7. Funnel plot for the effect of moderate prematurity/ low birthweight on cognitive function.


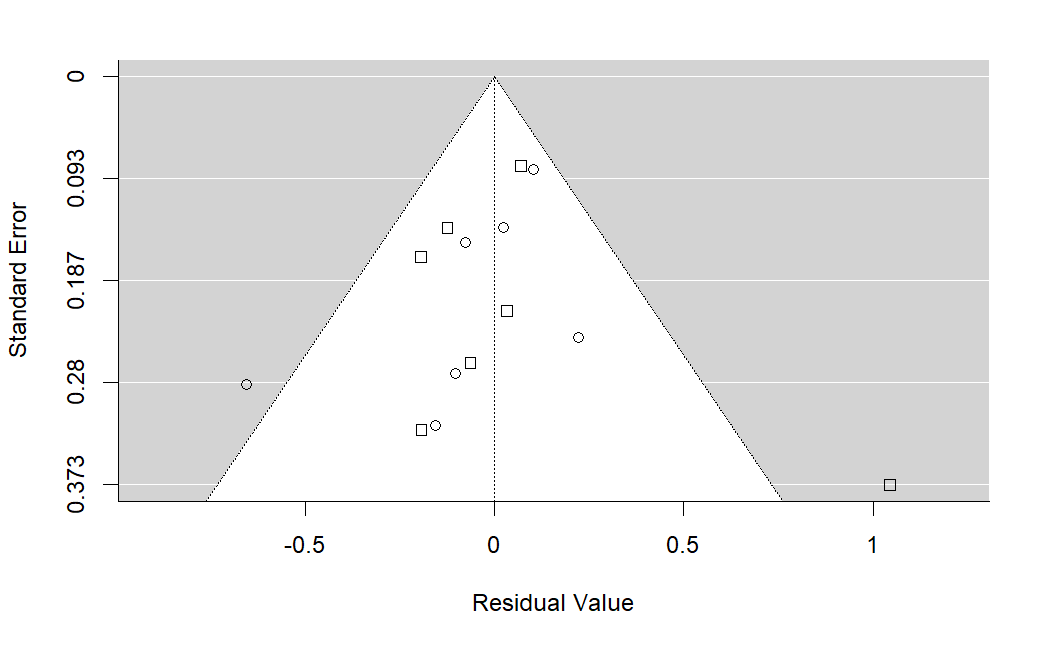


Figure S8. Funnel plot for the effect of severe prematurity/ low birthweight on internalizing problems.


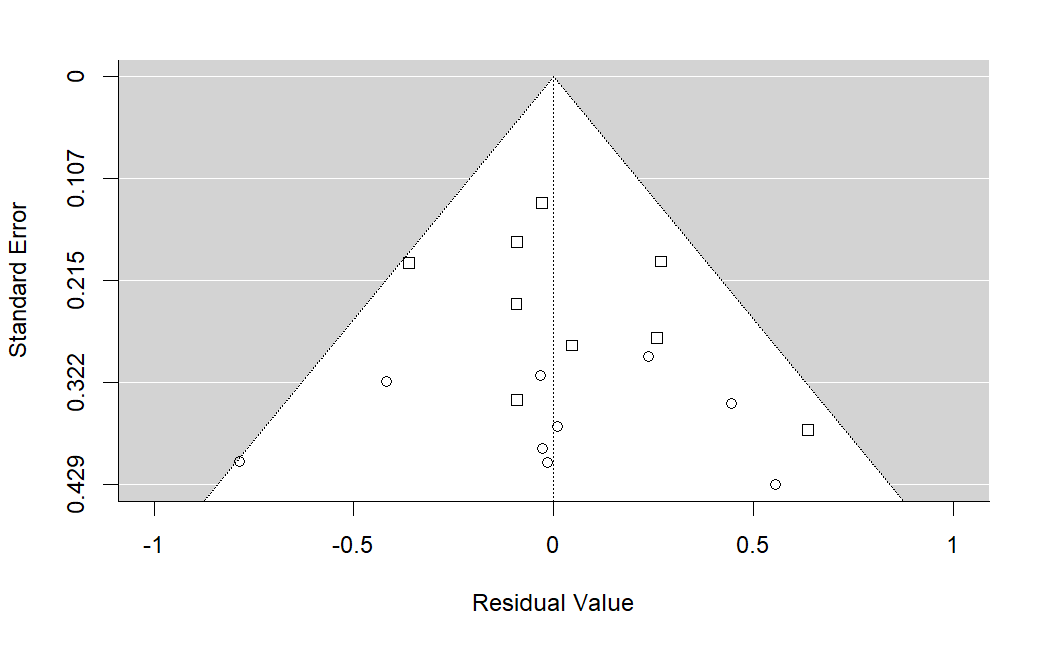


Figure S9. Funnel plot for the effect of severe prematurity/ low birthweight on externalizing problems.


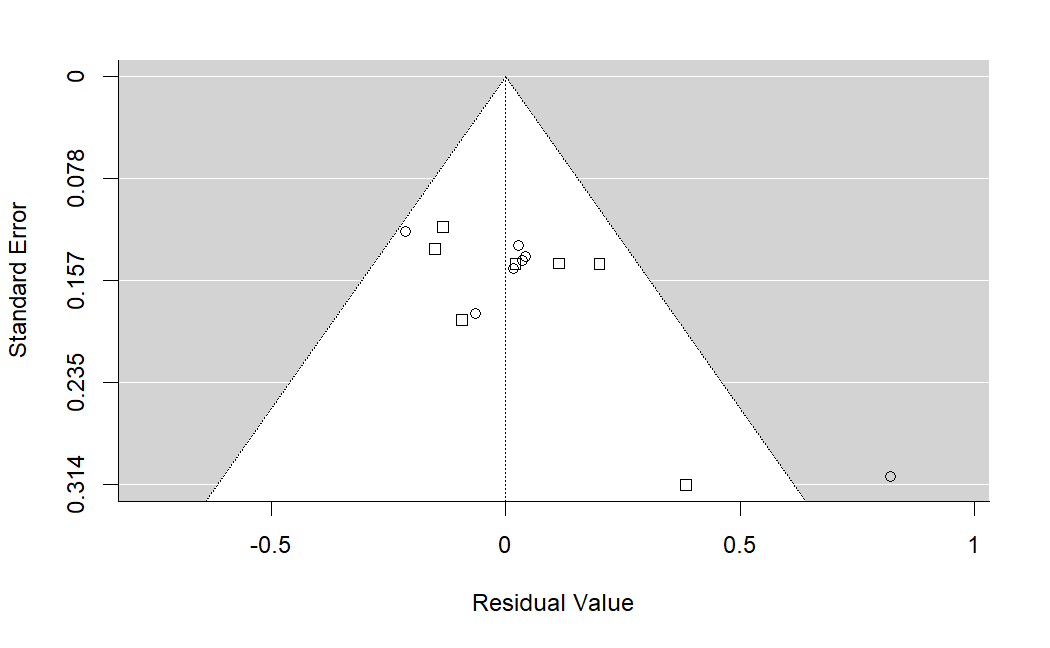


Figure S10. Funnel plot for the effect of moderate prematurity/ low birthweight on externalizing problems.
