# Supplementary material for: Sex differences in the effects of prematurity and/or low birthweight on neurodevelopmental outcomes: systematic review and meta-analyses
Source: Biol Sex Differ. 2023 Jul 11;14:47. doi: 10.1186/s13293-023-00532-9 (PMC10334669; doi:10.1186/s13293-023-00532-9)
Supplement: Supplementary file 4 — Additional file 4. Additional results. [file 13293_2023_532_MOESM4_ESM.docx]

**Additional Results**

*Quantitative synthesis by observer*

We repeated meta-analyses of internalizing and externalizing traits separately for self-reports and parent-reports. Because the combined analysis (in the main manuscript) averaged parent- and self-reported results for some studies, mean values listed in Figs S11-S15 do not always correspond to those in Figs 8-10.

*Effects of severe prematurity/ low birthweight on self-reported internalizing problem scores*

Five studies examined the effects of severe prematurity/ low birthweight on self-reported internalizing problem scores. Severe prematurity/ low birthweight did not affect self-reported problems scores (P = 0.35), and the estimated effect did not differ between males and females (P = 0.24; Figure S11). There was significant heterogeneity among studies (*I^2^* = 76%, *Q_E_* = 29, P = 0.0003).


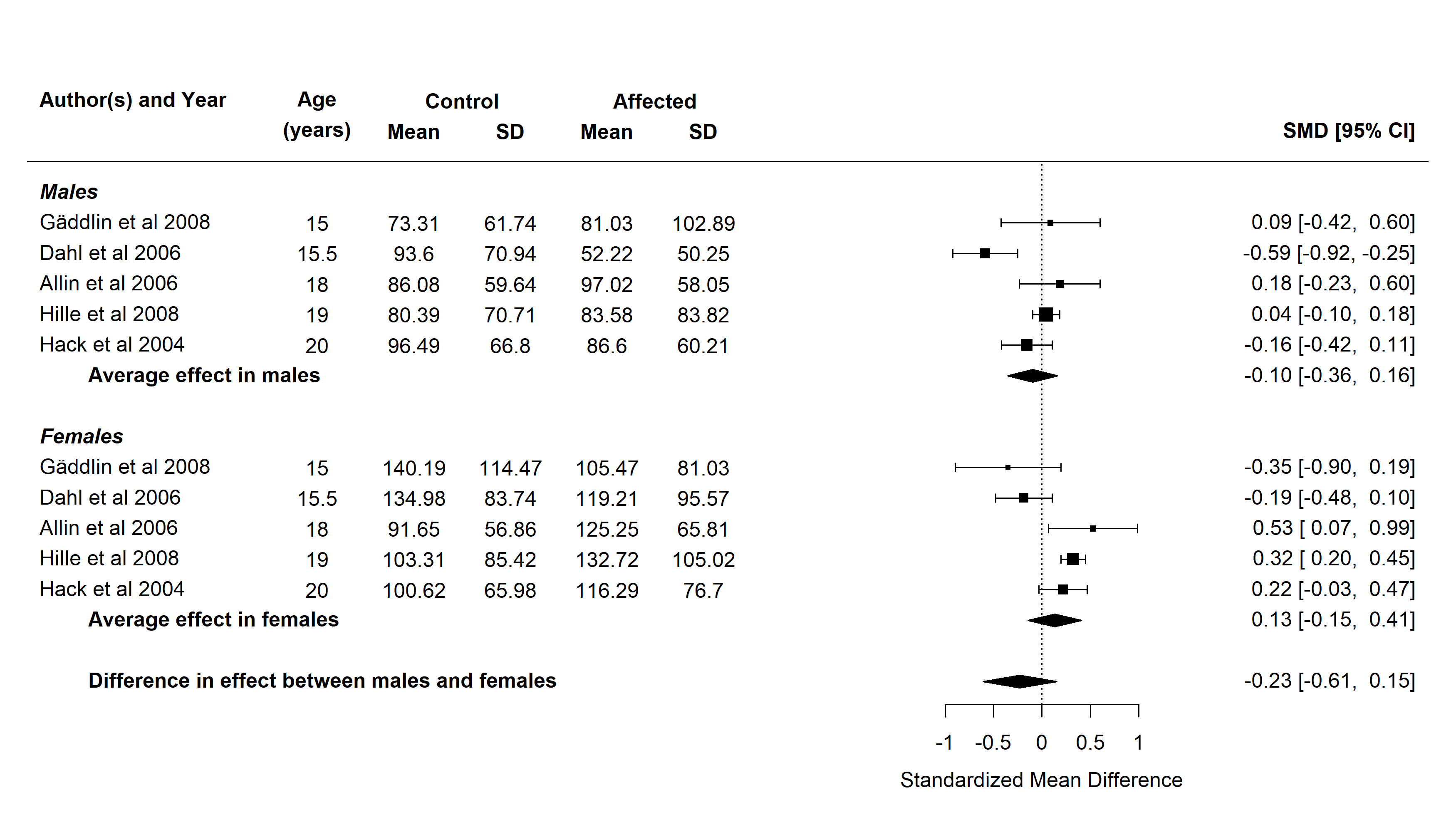


Figure S11. Meta-analysis of the effects of severe prematurity/ low birthweight on self-reported internalizing problem scores. Squares represent estimates (with confidence intervals) and marker size indicates weight. Diamonds represent estimates for each sex and for the difference between sexes, with the width of the diamond indicating the confidence interval. SMD: standardized mean difference.

*Effects of severe prematurity/ low birthweight on parent-reported internalizing problem scores*

Five studies examined the effects of severe prematurity/ low birthweight on parent-reported internalizing problem scores. Severe prematurity/ low birthweight increased parent-reported problems scores (P < 0.0001), but the estimated effect did not differ between males and females (P = 0.23; Figure S12). Heterogeneity among studies was not significant (*I^2^* = 0%, *Q_E_* = 11, P = 0.22).


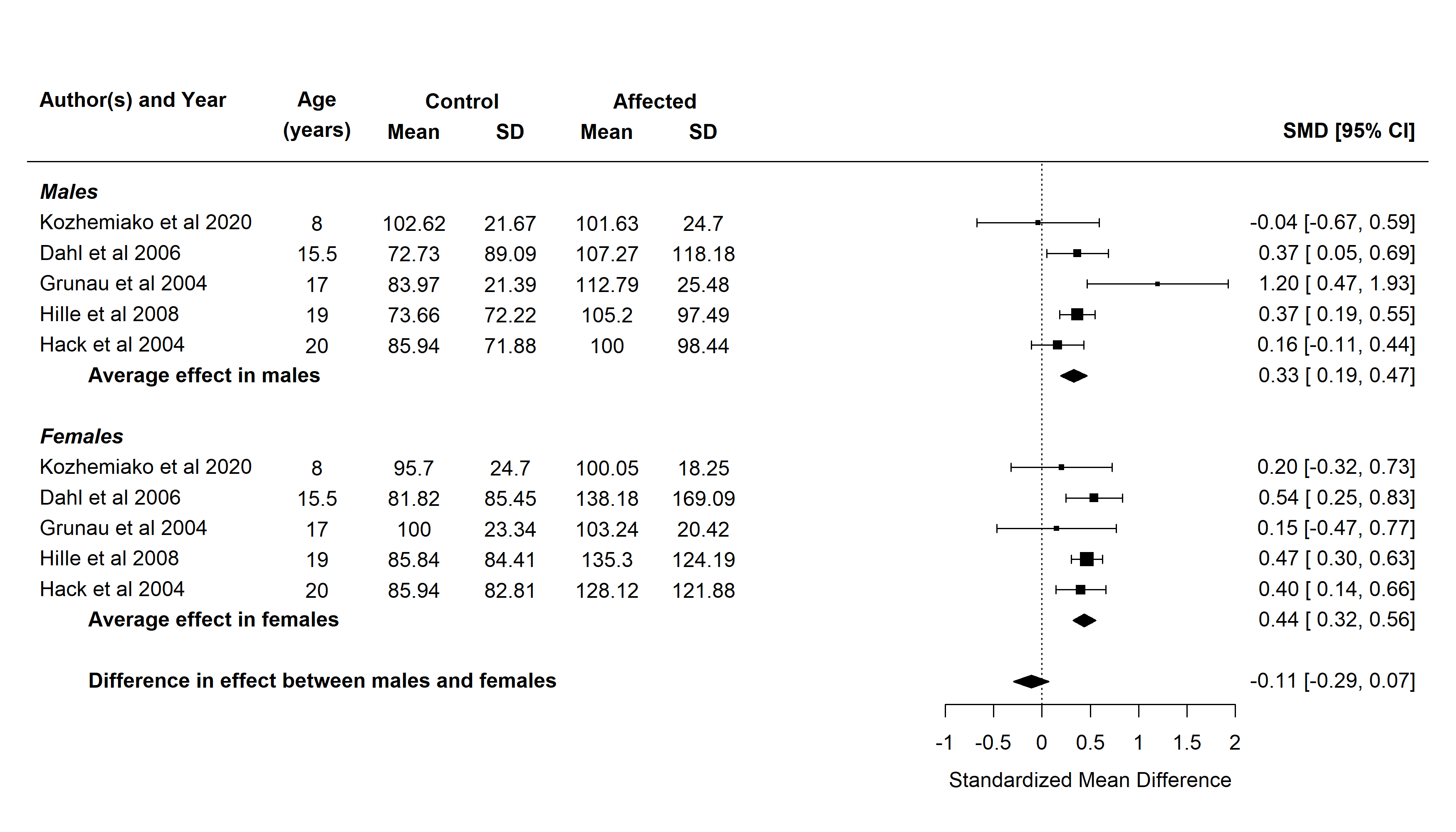


Figure S12. Meta-analysis of the effects of severe prematurity/ low birthweight on parent-reported internalizing problem scores.

*Effects of severe prematurity/ low birthweight on self-reported externalizing problem scores*

Five studies examined the effects of severe prematurity/ low birthweight on self-reported externalizing problem scores. Severe prematurity/ low birthweight did not affect self-reported problems scores (P = 0.10), and the estimated effect did not differ between males and females (P = 0.70; Figure S13). There was significant heterogeneity among studies (*I^2^* = 85%, *Q_E_* = 45, P < 0.0001).


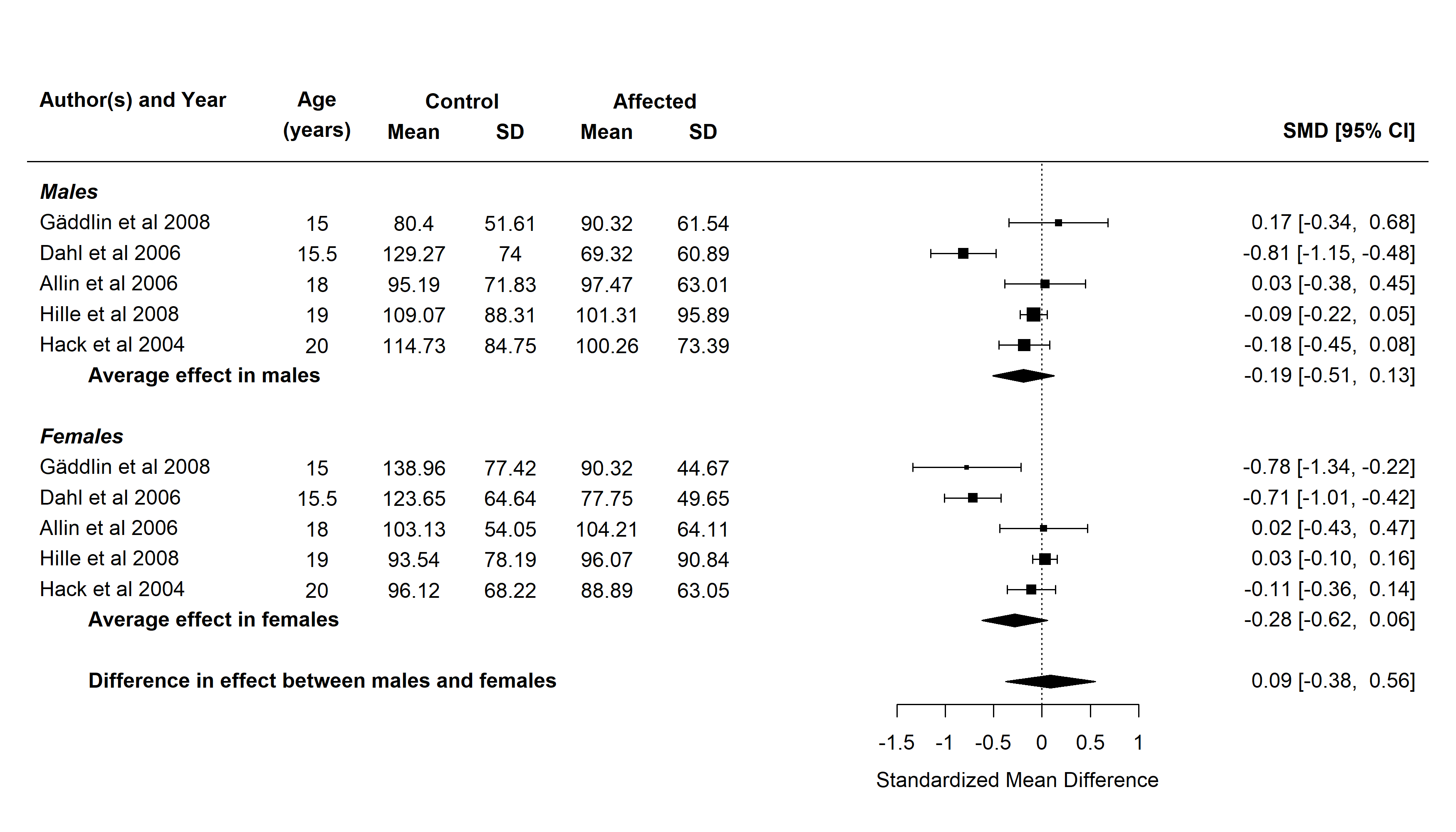


Figure S13. Meta-analysis of the effects of severe prematurity/ low birthweight on self-reported externalizing problem scores.

*Effects of severe prematurity/ low birthweight on parent-reported externalizing problem scores*

Seven studies examined the effects of severe prematurity/ low birthweight on parent-reported externalizing problem scores. Severe prematurity/ low birthweight did not affect parent-reported problems scores (P = 0.16), and the estimated effect did not differ between males and females (P = 0.48; Figure S14). There was significant heterogeneity among studies (*I^2^* = 46%, *Q_E_* = 22, P = 0.03).


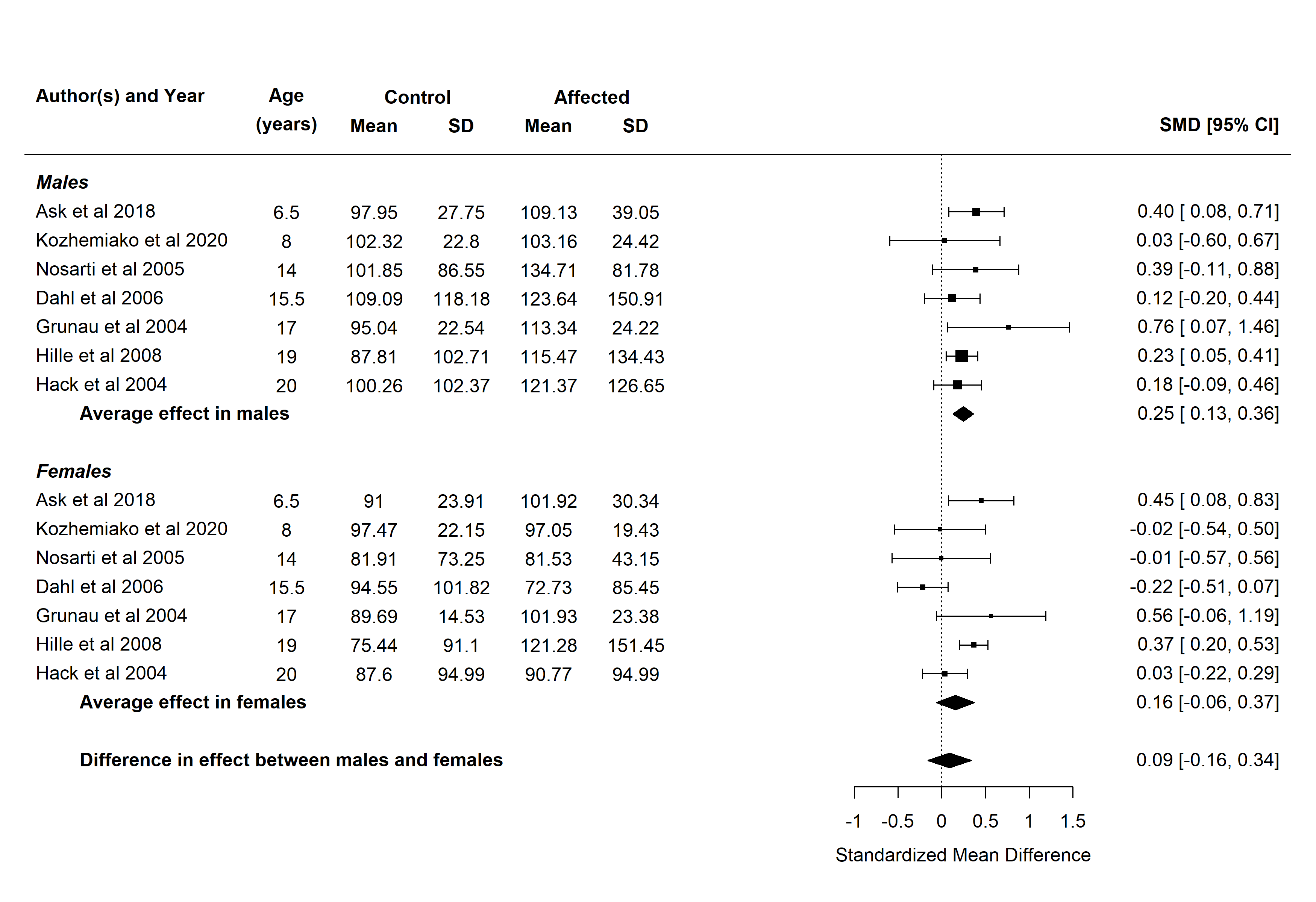
 Figure S14. Meta-analysis of the effects of severe prematurity/ low birthweight on parent-reported externalizing problem scores.

*Effects of moderate prematurity/ low birthweight on parent-reported externalizing problem scores*

Five studies examined the effects of moderate prematurity/ low birthweight on parent-reported externalizing problem scores. Severe prematurity/ low birthweight did not affect parent-reported problems scores (P = 0.06), and the estimated effect did not differ between males and females (P = 0.89; Figure S15). There was significant heterogeneity among studies (*I^2^* = 61%, *Q_E_* = 23, P = 0.004). There were no studies of the effects of moderate prematurity/ low birthweight on self-reported externalizing problem scores.


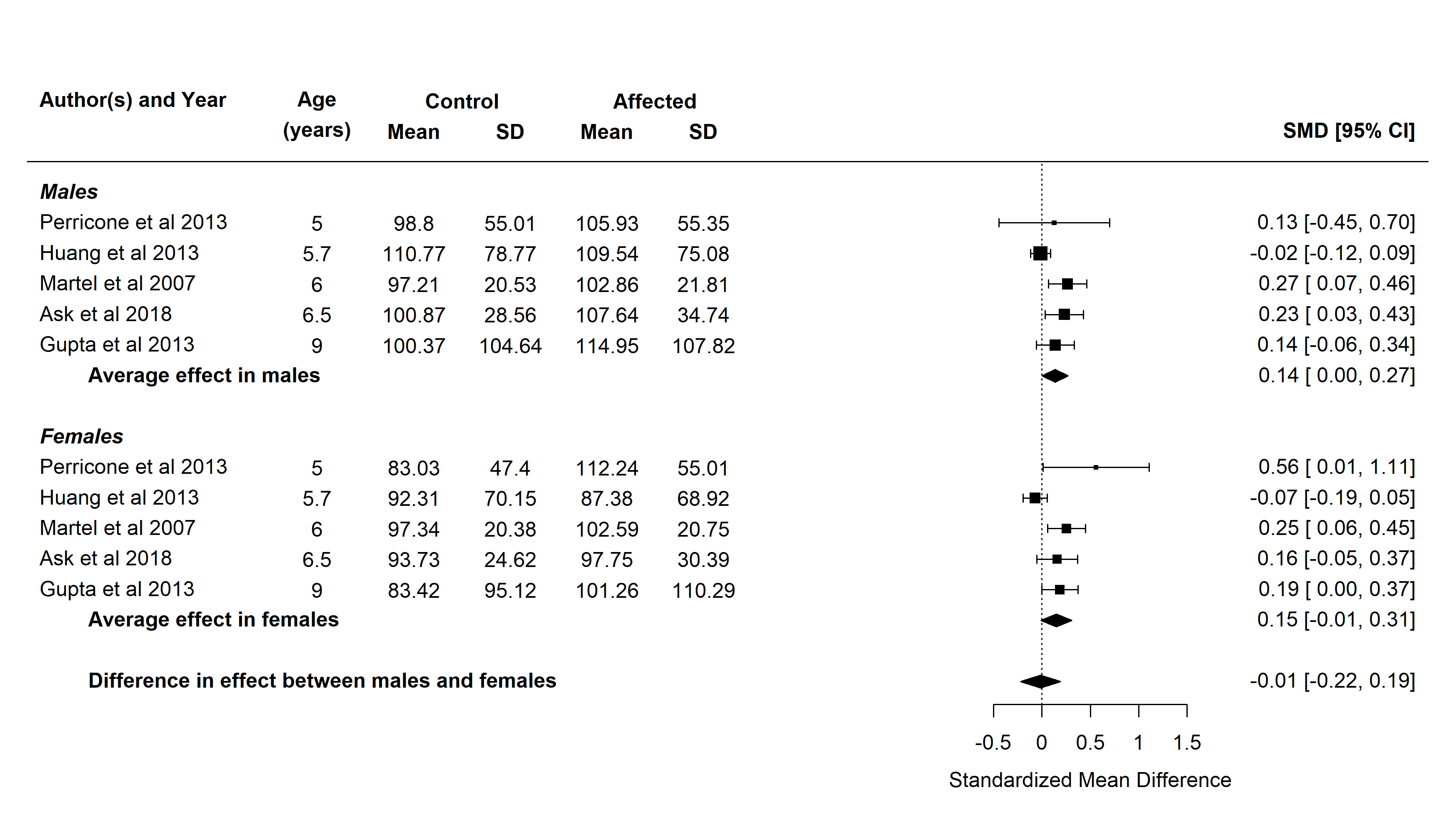


Figure S15. Meta-analysis of the effects of moderate prematurity/ low birthweight on parent-reported externalizing problem scores.
